# Spontaneous Modulation of Alpha Power During a Neurofeedback Session Without Instructions

**DOI:** 10.1111/psyp.70285

**Published:** 2026-03-24

**Authors:** Jacob Maaz, Véronique Paban, Laurent Waroquier, Arnaud Rey

**Affiliations:** ^1^ Aix Marseille Univ, CNRS CRPN Marseille France; ^2^ Institute Neuro‐Marseille Aix Marseille Univ Marseille France; ^3^ Institute of Language, Communication and the Brain Aix Marseille Univ Marseille France; ^4^ PSYCLE Aix Marseille Univ Marseille France

**Keywords:** alpha activity, EEG, neurofeedback, neuromodulation, spectral power

## Abstract

Electroencephalographic neurofeedback (EEG‐NF) enables individuals to self‐regulate specific EEG features with real‐time sensory feedback. Despite clinical and cognitive‐enhancement applications, the mechanisms underlying the EEG modulation remain poorly understood. Particularly, alpha activity (8–12 Hz) upregulation may occur independently of the participants' volitional control. We previously reported spontaneous increases in alpha power during a passive neurofeedback‐like task using pre‐recorded EEG feedback. In the present study, we replicated this protocol while implementing an EEG‐NF procedure using real‐time alpha power. Thirty‐two healthy adults observed a gray circle whose size was either fixed (control) or continuously updated at 1, 5 or 10 Hz (experimental conditions). Importantly, participants were not informed of the nature of the feedback and received no instructions for self‐regulation. We evaluated the effects of (i) trial repetition, (ii) the presence (control vs. experimental conditions), (iii) the frequency (1, 5 or 10 Hz) and (iv) the source (online vs. offline alpha) of feedback update on EEG features classically targeted by EEG‐NF. Importantly, we observed robust increases in alpha power over time independently of the presence, frequency and source of feedback update. The presence, frequency and source of feedback update did not influence the EEG features considered. These findings suggest that alpha EEG‐NF upregulation may arise from spontaneous dynamics, such as time‐on‐task effects, rather than the hypothesized self‐regulation mechanism. The assumption that observed alpha increases reflect successful neurofeedback learning is thus called into question. More broadly, the present study highlights the importance of including control conditions and accounting for non‐specific effects when evaluating EEG‐NF outcomes.

## Impact Statement

EEG neurofeedback often assumes that providing a sensory feedback of ongoing EEG activity allows individuals to volitionally control this activity. This study challenges this assumption by revealing that spontaneous EEG dynamics can mascaraed as successful EEG modulation typically targeted by EEG neurofeedback. These results call for rigorous control designs in neurofeedback research.

## 1 Introduction

Electroencephalographic neurofeedback (EEG‐NF) is a closed‐loop procedure in which individuals receive real‐time feedback of their own EEG activity and are encouraged to self‐regulate it (Sitaram et al. 2017). EEG‐NF modulates specific EEG features and is effective as both a clinical intervention and a training method to enhance cognitive functioning (Arns et al. 2017; Enriquez‐Geppert et al. 2017; Thibault et al. 2016). For instance, EEG‐NF reduces the symptoms of attention deficit and/or hyperactivity disorders (ADHD; Enriquez‐Geppert et al. 2019), depression and anxiety disorders (Hammond 2005), and pharmaco‐resistant epilepsy (Tan et al. 2009). Similarly, the cognitive performances of healthy individuals are improved in tasks involving memory, attention and procedural learning (for a review, see Gruzelier 2014a). Yet, despite decades of practice, skepticism persists on the actual mechanisms underlying the effectiveness of EEG‐NF on clinical/behavioral outcomes (Kalokairinou et al. 2022; Thibault and Raz 2017). Recent rigorous studies brought to light that these effects may actually be driven by non‐specific, confounded factors of the EEG‐NF procedure (Dessy et al. 2020; Neurofeedback Collaborative Group 2021, 2023; Schabus et al. 2017; Schönenberg et al. 2017). In particular, authors identify participants' expectations and motivation, and/or placebo effects (Dessy et al. 2018; Schönenberg et al. 2021; Thibault and Raz 2018).

Traditionally, the clinical and behavioral effects are specifically attributed to the EEG‐NF procedure and the targeted EEG modulation (Micoulaud‐Franchi et al. 2019). Accordingly, thanks to a real‐time sensory feedback, participants learn to regulate specific features of their EEG activity. In return, this self‐regulation reflects adaptive changes in brain function, which ultimately mediate the targeted therapeutic or behavioral effects (Chiasson et al. 2023). Thus, to expect an influence on behavior, one should first evaluate the success of the targeted EEG modulation. Classically, the EEG modulation is expressed in terms of EEG changes at three levels: within sessions (i.e., trial‐by‐trial), between sessions (i.e., longitudinal changes across training days), and by comparing EEG activity at rest before and after the EEG‐NF protocol (Ros et al. 2020). Yet, these forms of EEG modulation are inconsistently observed altogether in empirical studies, and there is no consensus on the most relevant outcome to evaluate it (Dempster and Vernon 2009; Egner et al. 2004; Mirifar et al. 2022; Schabus 2017, 2018; Witte et al. 2018). Moreover, there are substantial methodological variations between EEG‐NF protocols, including differences in feedback implementation, instructions, protocol length, trial structure, and whether or not a resting baseline is used to define positive feedback (Chiasson et al. 2023; Hasslinger et al. 2022; Muñoz‐Moldes and Cleeremans 2020). These discrepancies are currently not much considered in the field, although they may impact the EEG features targeted by the trainings (Strehl 2014). As such, it is not surprising that 15%–50% of EEG‐NF participants fail to modulate their EEG activity no matter the protocol (Alkoby et al. 2018; Kadosh and Staunton 2019; Wan et al. 2014). Overall, a common explanation of these issues may lie in the persistent lack of knowledge about the mechanisms underlying the targeted EEG changes in EEG‐NF protocols (Fovet et al. 2016). If one hopes to induce clinical/behavioral changes through the specific modulation of underlying EEG features, these mechanisms should be of main interest and better understood (Micoulaud‐Franchi and Fovet 2016).

Building on these current issues, we recently developed a passive EEG paradigm that mimicked the structure and environment of an EEG‐NF session, without involving real‐time EEG feedback nor explicit EEG self‐regulation instructions (Maaz et al. 2025). In this study, healthy participants were simply instructed, during multiple trials, to fixate on a gray circle that either remained unchanged (control condition) or was continuously updated in size, either at 1, 5 or 10 Hz (experimental conditions). The size variations were driven by pre‐recorded alpha band (8–12 Hz) power from a different group of participants, similarly to a sham feedback (Sorger et al. 2019). Results showed a spontaneous and consistent increase in frontal, central and parietal alpha power across trials, no matter if the circle was continuously updated or not. Additionally, an increase in theta power at Pz was observed when comparing conditions during which the stimulus was continuously updated relatively to the control, no‐update condition.

Importantly, such spectral changes in commonly targeted EEG‐NF features may, at least in part, non‐specifically explain the effects observed in genuine EEG‐NF sessions. Alpha, in particular, is considered as one of the most responsive spectral features to EEG‐NF self‐regulation (Belinskaia et al. 2020; Chikhi et al. 2023; Naas et al. 2019; Rogala et al. 2016). However, these recent findings suggest that the observed within‐session alpha upregulation through EEG‐NF is not solely elicited by its hypothesized self‐regulation by the participants (Maaz et al. 2025). Rather, it may be due to the non‐specific tendency of alpha power to increase with time‐on‐task (Arnau et al. 2020; Benwell et al. 2019; Kopčanová et al. 2025). Additionally, a very common practice to determine the positive feedback presented to the participant is to measure, prior training, a “baseline” of the targeted feature during a resting period without feedback (e.g., Agnoli et al. 2018; Gonçalves et al. 2018; Maszczyk et al. 2020). The theta increase triggered by the presentation of a continuously updated stimulus calls into question the validity of this approach. This emphasizes the necessity for the field to establish common evidence‐based standards to design EEG‐NF protocols (Chiasson et al. 2023; Strehl 2014).

The present study investigated whether the EEG power of frequency bands commonly targeted by EEG‐NF is influenced by: (i) the repeated presentation of the same visual stimulus over time (i.e., gray circle as pseudo visual feedback), (ii) the presence of a continuous feedback update within trials, (iii) the frequency at which the feedback is updated (1, 5 or 10 Hz), and (iv) the source of this feedback update. Particularly, the last point was evaluated by modifying the method used to generate the circle size variations in Maaz et al. (2025). Here, we implemented a visual display that matches a genuine alpha EEG‐NF session. As such, the circle size was updated in real time as a visual feedback of participants' own alpha power recorded at Pz. Importantly, participants were not instructed to self‐regulate their EEG activity, nor informed about the EEG‐driven nature of the feedback, resulting in a passive covert EEG‐NF paradigm (Muñoz‐Moldes and Cleeremans 2020). Accordingly, we aimed to assess whether the spontaneous EEG changes previously reported still occur in the presence of a closed‐loop feedback procedure, yet in the absence of explicit instructions for self‐regulation. We evaluated each effect and corresponding interactions on classically targeted EEG features in EEG‐NF research, that is, the spectral power of theta (4–8 Hz), alpha (8–12 Hz), sensorimotor rhythm (SMR; 12–15 Hz) and beta (15–30 Hz) frequency bands (Dessy et al. 2020; Micoulaud‐Franchi et al. 2015, 2019; Thibault et al. 2015). We hypothesized to replicate the findings of Maaz et al. (2025): (i) alpha power should spontaneously increase over time, no matter the presence, the frequency and the source of stimulus update (Benwell et al. 2019; Maaz et al. 2025), (ii) the continuous stimulus update should elicit an increase in theta power at Pz (VanRullen 2016), (iii) the frequency and (iv) the source of stimulus update should not influence any of the considered EEG features.

## 2 Method

### 2.1 Participants

Thirty‐two healthy young adults participated in the current study (*M*_age_ = 20.53 years, SD = 2.73, age range = 18–28; 25 females; 28 right‐handed [self‐reported]). Recruitment took place in November 2024 via Aix‐Marseille University's educational platform and the laboratory's communication channels. All participants were different from those enrolled in Maaz et al. (2025), reported having normal or corrected‐to‐normal vision and no history of neurological and/or psychiatric disorders. Twenty‐six participants were undergraduate students and received course credit in exchange for their involvement.

Written and informed consent was obtained from all participants in line with the Declaration of Helsinki. An anonymous code was assigned to each participant to ensure confidentiality. The study protocol was approved by the French Personal Protection Committee (CPP Sud Méditerranée V, ref. 19.09.12.44636).

### 2.2 Material and Feedback Implementation

The task consisted of an alpha EEG‐NF session with no explicit instructions to self‐regulate any EEG feature. The session included four conditions, each comprising eight 60‐s trials. During each trial, a gray circle was displayed in the centre of a blank screen. In the control condition, the circle size remained fixed throughout (radius of 100 pixels). In the other three experimental conditions, the circle size was updated in real time according to the spectral power of the alpha frequency band (8–12 Hz) at Pz (see the EEG online processing section for more information). Accordingly, during the trials of the experimental conditions, the circle size was positively proportional to the participant's alpha power at Pz computed in real time.

The feedback update was performed at one of three possible different frequencies, each of which corresponding to one experimental condition: 1 Hz, 5 Hz, or 10 Hz. These frequencies were selected based on their prevalence in EEG‐NF protocols with healthy adults (Berger and Davelaar 2018; Boe et al. 2014; Enriquez‐Geppert, Huster, Figge, and Herrmann 2014; Enriquez‐Geppert, Huster, Scharfenort, et al. 2014; Hsueh et al. 2016; Kober et al. 2015; Salari et al. 2014; Studer et al. 2014; Wei et al. 2017). Apart from the frequency at which the feedback was updated, all other circle features (color, maximum and minimum size) were held constant across trials and conditions.

To counterbalance the temporal order of conditions, a partial Latin‐square design was employed. For each participant, an in‐house Matlab script randomly selected one of four predefined quadruplets (1342, 2413, 3124 or 4231) as the condition order, while ensuring an even distribution across participants.

### 2.3 Apparatus

The EEG‐NF session and concurrent EEG data acquisition and online processing were implemented in Matlab Release 2023a (Mathworks Inc.) on a DELL Mobile 3571 computer with an Ubuntu 22.04 OS and a NVIDIA T600 Laptop GPU. Specifically, EEG data was acquired using the Brainflow library version 5‐8‐1 and an OpenBCI Cyton 8‐channels board with OpenBCI Gold cup and Earclip electrodes. Real time visual feedback was delivered via Psychtoolbox‐3 (Kleiner et al. 2007) and was displayed on a DELL P2419H flat‐screen monitor (resolution of 1920 × 1080 pixels; 60 Hz refresh rate; screen size of 52.704 × 29.646 cm). Participants were seated at a fixed distance of between 90 and 100 cm from the screen to the back of the chair, depending on individual seating needs. EEG offline processing was conducted in Matlab Release 2023a (Mathworks Inc.). Subsequent statistical analyses and figure generation were performed in R version 4.3.3 (R Core Team 2024).

### 2.4 EEG Recording

EEG was digitized using the OpenBCI board at a sampling rate of 250 Hz. EEG signals were recorded in microvolts (μV) in Matlab R2023a matrixes from six OpenBCI Gold Cup electrodes positioned at Fp1, Fpz, Fp2, Fz, Cz, and Pz (in accordance with the 10–20 International System). Two OpenBCI earclip electrodes were positioned on the left and the right earlobes to serve, respectively, as a reference for all electrodes and as a noise‐canceling ground electrode. Electrode impedance was maintained under 10 kΩ.

### 2.5 Procedure

Participants were seated in front of the monitor for the duration of the experiment. After obtaining written informed consent, the EEG setup was installed and impedance was checked. Participants were submitted to an alpha EEG‐NF session. Importantly, they were not informed that the circle size was actually a feedback of their alpha power. They were also not instructed to self‐regulate their EEG activity thanks to this feedback. Instead, participants were repeatedly asked to look at the circle presented on the monitor. Before the session began, the following verbal instructions were given in French: “You will complete 4 blocks of 8 trials, each lasting one minute. During each trial, a circle will be presented in the centre of the screen. During the 8 trials of a block, the circle can either remain the same, or its size will be changed continuously at the same rate. Your only task is to keep your eyes on the circle. There are no other particular instructions.” Participants were also encouraged to remain still and relaxed during trials to minimize artifacts in the EEG signal. Trials were initiated by pressing the “Enter” key on a keyboard positioned between the participant and the monitor. Participants were allowed to rest between trials at their own pace. At the end of the session, participants were invited to rate their feeling of control on the circle size variations based on a 5‐point Likert scale (from 1—“Not at all” to 5—“Totally”; see Table S1 for the repartition of responses among participants). Throughout the session, the experimenter (J.M.) stayed in the room but positioned out of the participant's view.

### 2.6 EEG Online Processing

During the trials of experimental conditions, the spectral power of the alpha frequency band (8–12 Hz) was computed in real time from Pz electrode. Alpha power was then used to continuously update the feedback presented to the participant (i.e., circle size). To this end, a time‐frequency analysis (moving window short‐FFT) was performed following the recommendations of (Keil et al. 2022; see Table S2 for the corresponding completed checklist). At each step, zero‐phase filtering was first performed with a 1–20 Hz bandpass filter (4th order IIR Butterworth) using the Matlab *filtfilt* function. Then, spectral power estimates were computed in decibels (*dB*) from a 2‐s (500 samples) symmetric Hann window using the Matlab *pspectrum* function. Alpha power was obtained by averaging spectral estimates within the 8–12 frequency range. Depending on the condition, the computation of alpha power have been done at different frequency rates to match the ones of the feedback update: 1 Hz, 5 Hz, or 10 Hz. For each experimental condition, the overlap between two consecutive analyzing windows has been determined accordingly by subtracting the analyzing window size (500 samples) by the EEG frequency sample (250 Hz) divided by the frequency of feedback update (1 Hz, 5 Hz, or 10 Hz). This respectively resulted in window overlaps of 250 (500–250/1), 450 (500–250/5) and 475 (500–250/10) samples (i.e., 50%, 90%, and 95%, respectively). To enable the feedback to be displayed from the start of each trial, EEG acquisition and the time‐frequency analysis began 3 s before the trial onset.

### 2.7 EEG Offline Processing

EEG data preprocessing and spectral analyses were conducted in line with the recommendations of (Keil et al. 2022; see Table S3 for the corresponding completed checklist). Custom Matlab scripts and the EEGLAB toolbox (Delorme and Makeig 2004) were used to extract spectral power in the theta (4–8 Hz), alpha (8–12 Hz), SMR (12–15 Hz) and beta (15–30 Hz) frequency bands. As neurofeedback research typically does not rely on explicit models of the oscillatory activity and the 1/*f* noise (Enriquez‐Geppert et al. 2017), we adopted the narrowband model for analyses (Keil et al. 2022). EEG preprocessing began with zero‐phase filtering applied on data from each electrode and trial. A high‐pass filter at 0.5 Hz (6th order IIR Butterworth) and a notch filter at 50 Hz (2nd order IIR) were implemented using the Matlab *filtfilt* function. This function avoids phase distortion by applying the filter forward and backward. Subsequently, filtered data was imported in EEGLAB, where an artifact correction was performed using the extended Infomax Independent Component Analysis (ICA; Delorme et al. 2007). ICA components associated with ocular artifacts (i.e., eye blinks and lateral eye movements) were visually identified and removed based on their scalp topography, time series, and power spectrum. One to three components were removed per participant (see Table S4 for details). The filtered and artifact‐corrected EEG data was exported back in Matlab format.

Subsequent analyses were restricted to data from Fz, Cz, and Pz electrodes. EEG signals were analyzed in the frequency domain using the Matlab *pspectrum* function. This function computes power spectra using FFT and Welch's method to enhance the robustness of spectral estimates. The function automatically determines the segment length to yield up to eight overlapping segments (50% overlap), which are each windowed with a Hamming function prior to FFT computation. The final spectral estimates reflect the average across these segments. For the current analyses, the frequency resolution applied was approximately 0.305 Hz. The spectral analyses were performed separately for each trial and participant and then converted to dB to favor a normal distribution of spectral power estimates. Finally, power estimates within each frequency band of interest (4–8 Hz, 8–12 Hz, 12–15 Hz, and 15–30 Hz) were averaged to obtain the spectral power of theta, alpha, SMR, and beta frequency bands, respectively.

### 2.8 Experiment of Maaz et al. (2025)

In Maaz et al. (2025), 32 healthy young adults underwent a highly similar experiment to that of the current study. Particularly, EEG acquisition and processing methods were identical. The full description of the rational, method, and results can be found in the original paper. The only difference between the two experiments lies in the source used for the continuous update of circle size during the experimental conditions. Compared to the alpha EEG‐NF procedure used in the current experiment, in Maaz et al. (2025)'s experiment, the circle size was continuously updated based on alpha power from previously recorded EEG data (i.e., similarly to a “sham” procedure; Thibault and Raz 2017). By comparing the present study to the one of Maaz et al. (2025), we hereby manipulate the source of feedback update (i.e., circle variations, either from ‘Online alpha’ for the current study, or ‘Offline alpha’ for Maaz et al. 2025).

### 2.9 Statistical Analyses and Hypotheses Testing

The resulting twelve dependent variables (i.e., spectral power of theta, alpha, SMR, and beta frequency bands measured by Fz, Cz, and Pz electrodes) were z‐score standardized across subjects of each experiment and used for statistical analyses. For the purpose of this study, we arbitrary hypothesized the presence of each effect of interest on these variables, that is, that their spectral power is influenced by (i) trial repetition, (ii) the continuous update of the feedback, (iii) the frequency of feedback update, and (iv) the source of feedback update.

To evaluate these effects, Bayesian linear multilevel modeling was used with the *brms* and *rstan* R packages (Bürkner 2017; Stan Development Team 2024). Bayesian methods offer several advantages over traditional frequentist approaches (Schad et al. 2021, 2023), including increased robustness to low‐power situations (Schönbrodt and Wagenmakers 2018), facilitated development and fit of multilevel models (Gelman et al. 2014), the possibility to distinguish sensitive from insensitive evidence for an absence of effect (i.e., the null hypothesis *H*_*0*_, Dienes and Mclatchie 2018), and the robustness to multiple comparison issues (Gelman et al. 2012). Each of the above mentioned dependent variables was considered as continuous in one model. Each model included the maximal varying effect structure to account for the individual variability of subjects (Barr et al. 2013). The Trial number continuous predictor (i.e., integers from 1 to 8), the Condition categorical predictor (i.e., Control, 1 Hz, 5 Hz, and 10 Hz), the Source categorical predictor (i.e., Online alpha, and Offline alpha), as well as their interaction were included in each model as fixed effects. For the Trial continuous predictor, we defined the first trial as a reference for comparison. For the Condition and Source categorical predictors, we followed Schad et al. (2020) guidelines to determine custom contrast matrixes for hypotheses testing via the generalized inverse (respectively presented in Tables S5 and S6). In particular, assigning a custom contrast matrix to the ‘Condition’ variable enabled us to test for an effect of both the continuous feedback update (i.e., Experimental conditions vs. Control) and the frequency of the feedback update (i.e., 5 Hz vs. 1 Hz, and 10 Hz vs. 5 Hz). To constrain the models to plausible values and to avoid overfitting issues, we placed regularizing priors of *N*(0, 1) on each parameter (Schad et al. 2021). Here is the full model equation (*brms* package syntax):

(1) $$\text{Power}\sim 1+\text{Trial}\times \text{Condition}\times \text{Source}+\left(1+\text{Trial}\times \text{Condition}\right|\text{Subject})$$

For each effect of interest, we computed Bayes Factors (BFs) to quantify the strength of evidence for an hypothesis over another (Dienes and Mclatchie 2018). To ensure we used enough Markov chain Monte Carlo (MCMC) draws to estimate stable BFs, we performed all reported statistical analyses three times (Schad et al. 2023). For each effect of interest, we report the mean of the three obtained posterior distributions, along with the largest limits of the 95% credible interval (CrI). We also report the mean of the obtained BF_10_ quantifying evidence for the presence of an effect (alternative hypothesis) over its absence (null hypothesis). As defined by Jeffreys (1939), we consider that a BF_10_ of above 3 indicates substantial evidence for the alternative over the null hypothesis, and that a BF_10_ of below ⅓ substantial evidence for the null over the alternative hypothesis. A BF_10_ between ⅓ and 3 indicates data insensitivity to distinguish null and alternative hypotheses (Dienes 2014). When an effect was confirmed (i.e., BF_10_ > 3), we also reported the BF_10+_ quantifying the amount of evidence for a positive‐directional (i.e., one‐sided) effect. Estimates (standardized units) and BFs from each of the models regarding the predictors of interest are presented in Table S8.

Finally, as we previously found an increase in alpha power over the whole course of the task of Maaz et al. (2025); no matter the conditions, we similarly evaluated in the longer run the time evolution of each frequency bands of interest. We reproduced the same statistical procedure and computed the same models (Equation 1) by excluding the Condition predictor and corresponding interactions. The Trial continuous predictor resulted in integers from 1 to 32 and the Source predictor remained the same. Estimates and BFs of these models are presented in Table S9. Here is the corresponding model equation (*brms* package syntax):

(2) $$\text{Power}\sim 1+\text{Trial}\times \text{Source}+\left(1+\text{Trial}\right|\text{Subject})$$

## 3 Results

### 3.1 Alpha Power Increases Over Time

**FIGURE 1:**
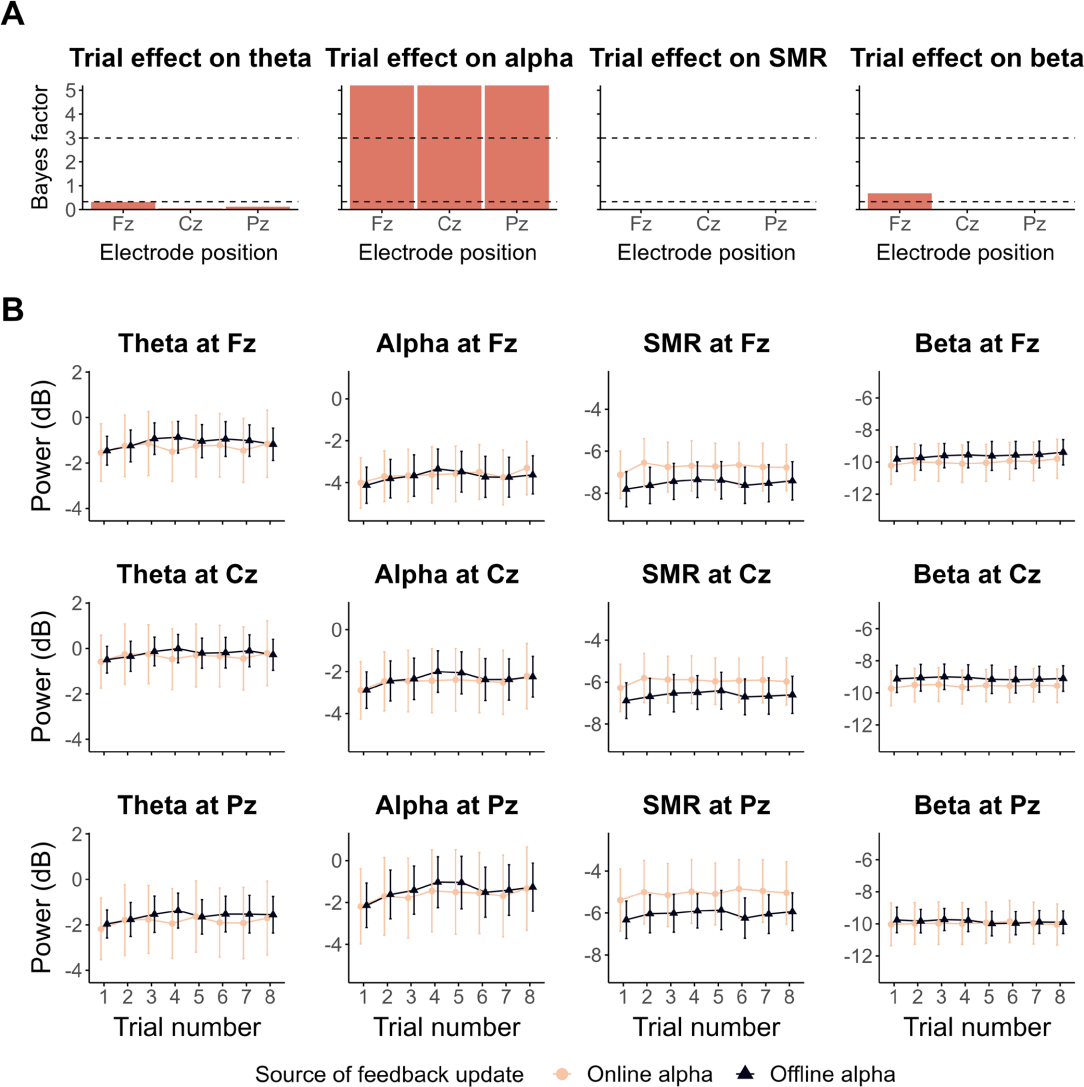
Trial repetition effect on EEG spectral power depending on the source of feedback update. (A) BF_10_ quantifying the evidence in favor of the presence, over the absence, of the trial repetition effect on theta (4–8 Hz), alpha (8–12 Hz), SMR (12–15 Hz) and beta (15–30 Hz) spectral power, respectively. On each plot, the dashed horizontal line at *y* = 3 indicates the level of substantial evidence in favor of an effect, whereas the dashed horizontal line at *y* = ⅓ indicates substantial evidence for no effect. (B) Evolution of theta, alpha, SMR, and beta spectral power (respectively across columns) at Fz, Cz and Pz (respectively across rows) across the trials of the control condition. Each line point represents the EEG spectral power averaged at the group‐level in function of the source of feedback update (light pink: Online alpha; dark blue: Offline alpha). Error bars indicate 95% confidence intervals.

Figure 1 shows how each frequency band (i.e., theta, alpha, SMR, beta) spectral power evolved over the eight trials of the control condition (in which the circle size remain constant) in both experiments (i.e., offline vs. online alpha). Within the control condition, we observed extreme evidence for a positive effect of trial repetition on the alpha spectral power at Fz (*β* = 0.016, 95% CrI [0.01, 0.022], BF_10_ > 100, BF_10+_ > 100), Cz (*β* = 0.017, 95% CrI [0.011, 0.023], BF_10_ > 100, BF_10+_ > 100) and Pz (*β* = 0.018, 95% CrI [0.011, 0.024], BF_10_ > 100, BF_10+_ > 100). These findings indicate that alpha power at Fz, Cz and Pz increases at each trial of the control condition (Figure 1D–F). For the other frequency bands of interest, we found substantial to extreme evidence for the absence of an effect (Figure 1A–C,G–I,K,L), except for the beta spectral power at Fz for which the BF_10_ was insensitive (*β* = 0.013, 95% CrI [0.005, 0.022], BF_10_ = 0.68; see Figure 1J). Table S7 reports the model estimates for the trial repetition parameter. Figure S1 shows the averaged power spectra depending on the trial number.

### 3.2 No Effect of the Continuous Update of Feedback, nor of the Frequency and of the Source of Feedback Update

**FIGURE 2:**
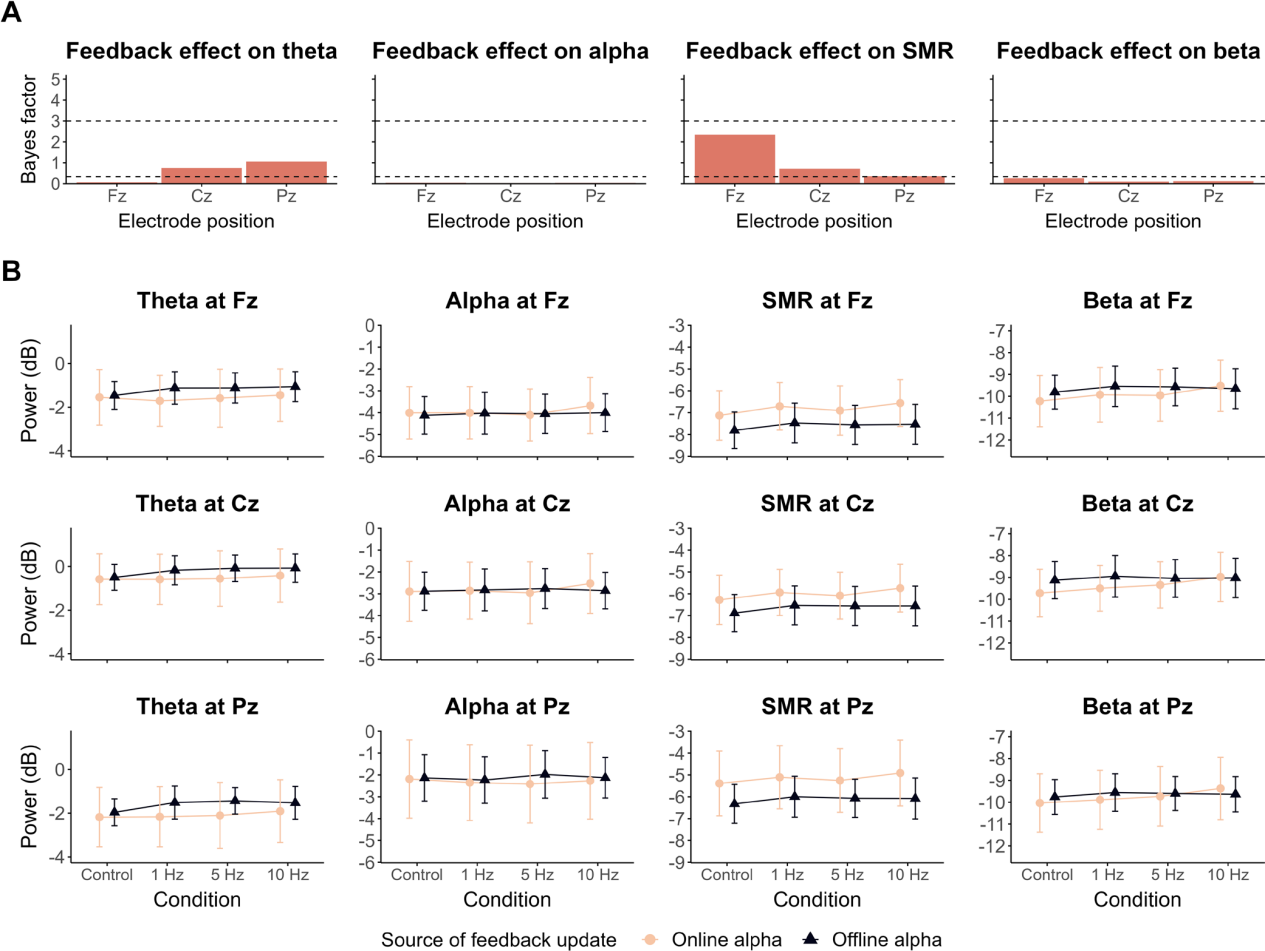
EEG spectral power at the first trial of each condition in both experiments. (A) BF_10_ quantifying the evidence in favor of the presence, over the absence, of the feedback update effect on theta (4–8 Hz), alpha (8–12 Hz), SMR (12–15 Hz) and beta (15–30 Hz) spectral power, respectively. On each plot, the dashed horizontal line at *y* = 3 indicates the level of substantial evidence in favor of an effect, whereas the dashed horizontal line at *y* = ⅓ indicates substantial evidence for no effect. (B) Spectral power of theta, alpha, SMR, and beta spectral power (respectively across columns) at Fz, Cz and Pz (respectively across rows) at the first trial of each condition. Each line point represents the EEG spectral power averaged at the group‐level in function of the source of feedback update (light pink: Online alpha; dark blue: Offline alpha). Error bars indicate 95% confidence intervals.

Figure 2 shows, depending on the source of feedback update, the spectral power of the considered frequency bands (i.e., theta, alpha, SMR, beta) during the first trial of each condition (i.e., Control, 1 Hz, 5 Hz and 10 Hz). When comparing the experimental conditions to the control (i.e., effect of the presence of feedback update), we found substantial to strong evidence supporting an absence of effect on theta power at Fz (Figure 2A), as well as on alpha (Figure 2D–F) and beta power (Figure 2J–L). Insensitive BF_10_ were obtained for the effect on SMR power (Figure 2G–I) at Fz (*β* = 0.091, 95% CrI [0.031, 0.152], BF_10_ = 2.338), Cz (*β* = 0.077, 95% CrI [0.017, 0.138], BF_10_ = 0.707) and Pz (*β* = 0.068, 95% CrI [0.007, 0.128], BF_10_ = 0.354), and on theta power (Figure 2B,C) at Cz (*β* = 0.074, 95% CrI [0.018, 0.131], BF_10_ = 0.755) and Pz (*β* = 0.081, 95% CrI [0.021, 0.141], BF_10_ = 1.059).

Concerning the effect of both the frequency and the source of feedback update, substantial to strong evidence supported the absence of an effect on most of the considered frequency bands (although see Figure S2 for visual entrainment effects of feedback update frequency). Exceptions concern the effect of the frequency of feedback update, in particular when comparing 10 to 5 Hz conditions for which insensitive evidence was obtained at Cz for SMR (*β* = 0.076, 95% CrI [0.01, 0.142], BF_10_ = 0.432) and beta power (*β* = 0.105, 95% CrI [0.022, 0.187], BF_10_ = 0.95).

Similarly, for all interactions effects, we found substantial to extreme evidence for no effect on most of the considered frequency bands. The only exceptions concern the interaction between the feedback update and the source of feedback update, quantifying insensitive evidence for the effect on theta at Cz (*β* = 0.074, 95% CrI [0.017, 0.131], BF_10_ = 0.713) and Pz (*β* = 0.085, 95% CrI [0.025, 0.145], BF_10_ = 1.457). Overall, these findings suggest that alpha power increases over time, regardless of whether feedback is updated (Experimental conditions vs. Control), how frequently it is updated (1, 5, or 10 Hz), or its source (online vs. offline alpha). All the model estimates and BFs of the parameters reported in this section are presented in Table S8.

### 3.3 Alpha Increase Is Maintained Throughout the Session no Matter the Source of Feedback Update

**FIGURE 3:**
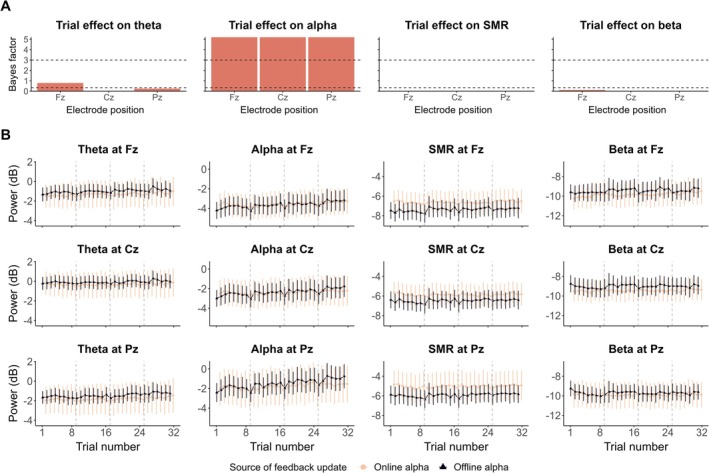
Evolution of EEG spectral power throughout the session. (A) BF_10_ quantifying the evidence in favor of the presence, over the absence, of the trial repetition effect (throughout the session) on theta (4–8 Hz), alpha (8–12 Hz), SMR (12–15 Hz) and beta (15–30 Hz) spectral power, respectively. On each plot, the dashed horizontal line at *y* = 3 indicates the level of substantial evidence in favor of an effect, whereas the dashed horizontal line at *y* = ⅓ indicates substantial evidence for no effect. (B) Evolution of theta, alpha, SMR, and beta spectral power (respectively across columns) at Fz, Cz, and Pz (respectively across rows) throughout the session. Each line point represents the EEG spectral power averaged at the group‐level in function of the source of feedback update (light pink: Online alpha; dark blue: Offline alpha). Error bars indicate 95% confidence intervals. Vertical dashed lines mark the first trial of each condition.

In this section, we report the results of the models taking into account the trial repetition effect over the entire session, no matter the condition (Equation 2). Figure 3 shows the evolution, depending on the source of feedback update, of the considered frequency bands (i.e., theta, alpha, SMR, and beta) over the 32 trials of the experiment. We found extreme evidence that the alpha power at Fz (*β* = 0.007, 95% CrI [0.004, 0.01], BF_10_ > 100, BF_10+_ > 100), Cz (*β* = 0.007, 95% CrI [0.004, 0.01], BF_10_ > 100, BF_10+_ > 100), and Pz (*β* = 0.009, 95% CrI [0.006, 0.012], BF_10_ > 100, BF_10+_ > 100) increased throughout the entire session in both experiments (Figure 3D–F). Substantial to extreme evidence supported the absence of trial repetition effect on the remaining frequency bands (Figure 3A,B,G–L), except for the theta power at Pz for which the amount of evidence was insensitive (*β* = 0.006, 95% CrI [0.003, 0.009], BF_10_ = 0.792; see Figure 3C). Model estimates are presented in Table S9.

Concerning the effect of the source of feedback update, as well as the interaction with trial repetition, we obtained substantial to extreme evidence of no effect on all considered frequency bands, except for the interaction effect on alpha power at Pz (*β* = −0.005, 95% CrI [−0.007, −0.002], BF_10_ = 0.473). Overall, these results indicate that, throughout the entire session, the source of feedback update does not influence the power of theta, alpha, SMR, and beta frequency bands. More importantly, the increase in alpha power over time is observed no matter the source of feedback update.

## 4 Discussion

The present study aimed to assess whether spontaneous EEG changes observed in a previous pseudo‐neurofeedback task (Maaz et al. 2025) would replicate in a genuine EEG‐NF session using real‐time alpha feedback, but without explicitly engaging participants into its self‐regulation. We investigated whether the spectral power of commonly targeted frequency bands in EEG‐NF (i.e., theta, alpha, SMR, and beta) was influenced by the repeated presentation of a visual stimulus, the presence of its continuous update, the frequency and the source of this update (online vs. offline alpha power). In line with our hypotheses and previous results, we found that alpha power increased across trials at all electrode sites, regardless of the presence, frequency, or source of the feedback update. However, we did not replicate the previous finding of increased theta power at Pz relative to the presence of a continuously updated visual stimulus. No other effects were found for SMR or beta power.

When assessing the influence of the source of the visual feedback update (online alpha in the current experiment vs. offline alpha in Maaz et al. 2025) on the alpha power increases, results revealed no effect of the source of feedback update, as well as an absence of interaction with trial repetition. Importantly, the same conclusions were drawn when analyzing the alpha evolution over the whole session, without taking into account the Condition predictor (Equation 2; see Figure 3D–F). Thus, in EEG‐NF settings, alpha power tends to exhibit a positive non‐stationarity which cannot be attributed to the visual stimulus presented, its continuous update over time, the frequency of this update nor whether it is EEG‐driven or not.

The alpha band is one of the EEG features most frequently targeted in EEG‐NF history (Belinskaia et al. 2020; Micoulaud‐Franchi et al. 2019; Nowlis and Kamiya 1970). Alpha activity has been widely linked with relaxation and meditation (Bing‐Canar et al. 2016; Magosso et al. 2019). Alpha upregulation through EEG‐NF has been observed within and between EEG‐NF sessions (Belinskaia et al. 2020; Chikhi et al. 2023, 2024; Dempster and Vernon 2009; Escolano et al. 2014; Grosselin et al. 2021; Naas et al. 2019; Nowlis and Kamiya 1970; Su et al. 2021; Zoefel et al. 2011), as well as during resting‐state EEG before and after EEG‐NF training (Dekker et al. 2014; Lee et al. 2019). Consequently, this effective modulation is considered as responsible for alpha EEG‐NF effectiveness as a non‐pharmacological treatment of post‐traumatic stress disorders (PTSD; Kluetsch et al. 2014), depression and anxiety (Hardt and Kamiya 1978; Peeters et al. 2014), headaches (Stokes and Lappin 2010) and even tinnitus (Jensen et al. 2023). In healthy individuals, alpha modulation is also targeted to enhance memory (Naas et al. 2019; Yeh et al. 2021), attention (Bagherzadeh et al. 2020; Berger and Davelaar 2018) and creative (Agnoli et al. 2018) performances.

In the current study, the alpha power increased in a situation where participants were not explicitly engaged in any EEG self‐regulation (i.e., passive feedback visualization). Still, they were provided with genuine sensory feedback of their real‐time alpha power. These results are particularly congruent with previous alpha upregulation in the studies of Dekker et al. (2014) and van Boxtel et al. (2012). During fifteen consecutive EEG‐NF daily‐sessions, healthy participants were provided with an alpha auditory feedback. Importantly, participants were not explicitly instructed to self‐regulate their alpha thanks to the feedback, as in the current study. Yet, alpha increases were observed within and between EEG‐NF sessions, as well as at rest before and after training. These results were interpreted as successful alpha upregulation through EEG‐NF, suggesting that the mere presentation of a sensory feedback is sufficient to drive the targeted EEG modulation. In that case, instructing participants to self‐regulate their EEG activity would not be necessary.

However, alpha power is also known to elicit upward non‐stationarities over time, independently of any experimental manipulation (Benwell et al. 2019; Kopčanová et al. 2025; Maaz et al. 2025). As a result, without properly controlling for these non‐specific influences, one cannot confidently conclude that the observed alpha evolution during genuine EEG‐NF alone reflects successful EEG modulation. Here, we systematically compared the alpha trajectories in the present genuine EEG‐NF settings with an independent group (Maaz et al. 2025). This group underwent almost identical testing to that of the present group. They were repeatedly presented with either a fixed or a continuously modified circle, without explicit instructions to self‐modulate their EEG activity thanks to the circle variations. The only between‐group difference was the source of feedback update, that is, real‐time alpha power at Pz for the current study and prerecorded alpha trajectories for Maaz et al. (2025). Critically, Bayesian analyses confirmed the absence of any effect of the source of feedback update on alpha power, as well as an absence of interaction with trial repetition. These findings suggest that the present and previously observed alpha power increases during EEG‐NF (Dekker et al. 2014; van Boxtel et al. 2012) may occur independently of a genuine closed‐loop feedback procedure. Rather, previously acknowledged alpha non‐stationarities most likely drove, non‐specifically, the targeted alpha upregulation (Maaz et al. 2025).

To control non‐specific influences on both EEG and clinical/behavioral outcomes, sham EEG‐NF protocols are considered as the gold standard control condition/group (Ros et al. 2020; Thibault and Raz 2017; although see Sorger et al. 2019 for a comprehensive review highlighting alternative controls). Everything else being equal, the only difference between sham and genuine EEG‐NF protocols is the presence or absence of a link between the sensory feedback presented to the participants and the targeted EEG feature(s). In sham protocols, without informing the participant, the sensory feedback is disconnected from the targeted EEG feature(s), which rules out the possibility of self‐regulation (Sorger et al. 2019). Therefore, if EEG modulation occurs through the hypothesized self‐regulation mechanism, genuine EEG‐NF protocols should produce superior EEG changes (Thibault and Raz 2018). However, as the entire field, alpha EEG‐NF studies do not systematically implement a sham (or even an active control) condition/group (Chikhi et al. 2023; Dekker et al. 2014; Lee et al. 2019; Yeh et al. 2021; Zoefel et al. 2011). While some exceptions exist (Escolano et al. 2014; Grosselin et al. 2021), alpha increases has also been observed independently of its possibility to be trained (Belinskaia et al. 2020; Dessy et al. 2020; Jiang et al. 2021; Naas et al. 2019). These observations align with the present results. By manipulating the source of feedback update, this study ensured that the targeted alpha upregulation was effectively due to the presence of a closed‐loop feedback procedure. To this end, the sample from Maaz et al. (2025) was used as a sham‐like control group. Overall, these findings highlight the necessity for EEG‐NF to control non‐specific influences that could be mistaken for successful EEG modulation.

Furtherly, while sham EEG‐NF is considered as a gold‐standard control, both genuine and sham protocols comprise a variety of factors which can influence EEG activity. Typically, sham protocols are designed to control for the influence of non‐specific cognitive (engagement in self‐regulation), psychosocial (motivation, interaction with the practitioner, expectations, general neurotechnological context) or general repetition‐related factors confounded in the genuine EEG‐NF procedure (Thibault and Raz 2018). By comparing genuine and sham protocols, one can consequently infer whether the observed effects are due to EEG self‐regulation or to non‐specific cognitive, psychosocial and/or repetition‐related factors. As such, EEG changes have been reported to occur in sham protocols (e.g., Belinskaia et al. 2020; Naas et al. 2019; Schabus et al. 2017). However, when using sham as a stand‐alone control, the cognitive, psychosocial or repetition‐related nature of these non‐specific influences remain to be determined. By additionally using a similar control group to the one of the present study, one can distinguish between non‐specific influences related to time‐on‐task to higher‐level cognitive and psychosocial factors confound in the sham procedure.

Importantly, the question of non‐specific repetition‐related influences is particularly relevant for alpha EEG‐NF. Outside of EEG‐NF context, alpha activity is considered as an active mechanism for inhibiting or gating sensory input, playing a central role in attention and memory processes (Klimesch 1999, 2012; Klimesch et al. 2007). More broadly, alpha is known as exhibited versus inhibited depending on the internal (i.e., self‐focused) versus external (i.e., environment‐focused) focus of attention, respectively (Hanslmayr et al. 2011; Sauseng et al. 2005; Wang et al. 2016). Unsurprisingly, alpha activity is enhanced during episodes of mind wandering, defined as a switch of attention towards internal thoughts unrelated to the task in hand (Arnau et al. 2020; Compton et al. 2019). In addition, the occurrence of mind wandering increases with time‐on‐task (Compton et al. 2024; Gouraud et al. 2018). Thus, the present increases in alpha power over time, as in broader cognitive tasks (Arnau et al. 2020, 2021; Benwell et al. 2019), might be elicited by an increasing internal focus of attention during mind wandering episodes. As a result, this phenomenon may, at least in part, explain the EEG changes in terms of enhanced alpha power in both sham and genuine EEG‐NF protocols (e.g., Belinskaia et al. 2020).

Moreover, this study investigated whether presenting a continuously updated visual stimulus (vs. presenting the same unmodified stimulus over time) has an influence on the spectral power of frequency bands classically targeted by EEG‐NF (i.e., theta, alpha, SMR, and beta). This question is highly relevant given the current lack of methodological standards in the field when designing EEG‐NF protocols (Chiasson et al. 2023; Gruzelier 2014b; Hasslinger et al. 2022; Strehl 2014). In particular, to determine when to provide positive feedback to participants (i.e., indication of success in self‐modulation at a given time point), a common practice is first to measure the targeted EEG feature during a resting‐state “baseline”, during which participants usually fixate passively on a cross. During training, if the real‐time computed EEG feature exceeds a threshold determined by its baseline value, positive feedback is given to the participant (e.g., Agnoli et al. 2018; Enriquez‐Geppert et al. 2017; Gonçalves et al. 2018; Maszczyk et al. 2020). However, this approach can be problematic, since EEG‐NF training involves active participant engagement in self‐regulation, unlike resting‐state baseline measures. This engagement can influence the targeted EEG feature through the involvement of higher cognitive processes (Gaume et al. 2016; Micoulaud Franchi et al. 2020). Additionally, the perception of a continuously modified stimulus can alter EEG spectral densities, in particular in the theta and alpha frequency bands (e.g., Spaak et al. 2014; VanRullen 2016). If so, the determination of positive feedback during training may be biased, resulting in an unclear evaluation of the subsequent EEG modulation. In our previous work, we found that the presentation of a continuously updated visual stimulus, compared to no modification, elicits an increase in theta power at Pz (Maaz et al. 2025). We therefore highlighted the need to change the current method to determine positive feedback in threshold‐based approaches, in particular for protocols focusing on theta upregulation at Pz (e.g., Egner and Gruzelier 2004; Rozengurt et al. 2016). Surprisingly, we did not replicate this finding in the current study and we cannot actually conclude about the presence or absence of an effect. Indeed, the BF_10_ evaluating the effect of the continuous stimulus update and the corresponding interaction with the source of feedback update (i.e., the between‐group predictor comparing the two studies) quantified insensitive amount of evidence (see Table S8; Dienes and Mclatchie 2018; Jeffreys 1939).

The failure to replicate this finding could be explained by the main difference between the two studies, that is, the source of feedback update. In the present study, the feedback update was based on the online‐computed alpha power of the participant, that is, similarly to a genuine EEG‐NF procedure. By contrast, Maaz et al. (2025) used pre‐recorded alpha power from a pilot study. Specifically, for each trial during which the circle size was updated, each circle size variation was determined in real time by sampling at random one possible variation of alpha power pre‐recorded during the pilot. Yet, while successfully providing circle size variations disconnected from participant EEG activity, this method yielded to suboptimal representation of alpha temporal dynamics. Therefore, this mismatch in alpha temporal dynamics between studies might explain the divergent effects on theta, which may be result of distinct visual information processing.

Here, in addition to the non‐replication of the effect of the continuous feedback update, we mostly confirmed the absence of influence from either the frequency and the source of feedback update on EEG features classically targeted by EEG‐NF (for few exceptions, for which we found insensitive evidence, see Tables S8 and S9). We would like to emphasize the reassuring nature of these findings for the field. Indeed, these effects addressed important methodological issues which did not receive enough consideration so far (Chiasson et al. 2023; Hasslinger et al. 2022; Witte et al. 2018). In particular, the frequency of feedback update is not currently standardized in the field. Whether at 1, 5 or 10 Hz, this frequency may influence the timing of the learning process occurring during EEG‐NF sessions (Belinskaia et al. 2020; Fingelkurts and Fingelkurts 2006; Michel and Koenig 2018). Similarly, whether genuine or sham, the source of feedback update influences per se the learning process (Pigott et al. 2017, 2018). The present results suggest that, at least perceptually, the update, the frequency and the source of feedback do not influence EEG features typically targeted. However, contrary to sham and genuine EEG‐NF protocols, the present experiment lacked explicit instructions for participants to engage in EEG self‐regulation. As such, the extent to which each of the manipulated factors influences EEG features during actual EEG‐NF settings (i.e., with explicit instructions for self‐regulation) remains to be determined by further studies.

Overall, the present study provides valuable methodological considerations for EEG‐NF protocols, being reassuring especially concerning the feedback generation procedure (no effect of the presence, the frequency nor the source of feedback update). More importantly, however, the current increase in alpha power over time, which was also reported in a similar context (Maaz et al. 2025), contributes to the broader discussion about the mechanisms underlying EEG‐NF (Micoulaud‐Franchi and Fovet 2018; Thibault and Raz 2017, 2018). Specifically, alpha increases occurred independently of the continuous update of the feedback (control vs. experimental conditions), its frequency rate (whether at 1, 5 or 10 Hz) and its EEG‐driven nature (i.e., source of feedback update). As such, the present increases in alpha power match the two criteria to reflect spontaneous activity, that is, not being influenced by the feedback display in a situation during which participants are not activity engaged in the self‐regulation task (Ramot and Martin 2022). While the targeted EEG modulation is traditionally framed as resulting from a self‐regulation mechanism (Gaume et al. 2016; Mirifar et al. 2022), the current study suggests that spontaneous EEG dynamics can mimic the patterns expected from successful training in the absence of intentional modulation. This aligns with critiques emphasizing the need to distinguish true self‐regulation from time‐related EEG changes, placebo effects, and protocol artifacts (Schabus 2017, 2018; Witte et al. 2018). More generally, the present data call for caution in interpreting EEG‐NF outcomes without rigorous control conditions, in particular concerning protocols targeting alpha upregulation. Further empirical studies aiming to characterize the EEG modulation underlying mechanisms are encouraged.

Specifically, a direct comparison of the current alpha increase with the alpha evolution during both genuine EEG‐NF and a proper control protocol would help to isolate the mechanisms responsible for alpha modulation (i.e., specific self‐regulation, non‐specific cognitive and/or psychosocial factors, and/or general non‐specific effects such as time‐on‐task). In terms of control protocols, several possibilities have been previously acknowledged (Sorger et al. 2019). In particular, above‐mentioned sham EEG‐NF would enable to conclude whether specific self‐regulation, acquired through the repeated exposure to genuine feedback, can overcome or equals non‐specific influences in terms of EEG changes. Furthermore, comparing an active and a passive sham protocols would enable to dissociate between repetition‐related and more higher cognitive/psychosocial non‐specific influences (Maaz et al. 2025). In particular, a relevant extension of the present results would imply to directly compare the current spontaneous alpha increases in passive settings with alpha trajectories during an active EEG‐NF protocol (i.e., with participants being explicitly engaged in the self‐regulation procedure). In addition, alternative protocols such as bidirectional control (i.e., manipulating the direction of targeted EEG modulation) can powerfully account for non‐specific influences while also enabling a direct evaluation of self‐regulation occurrence.

Lastly, the present study encompasses two main limitations that constrain the time‐on‐task interpretation of alpha increases. First, the current experimental design did not incorporate direct artifact‐level metrics, such as eye blink rate over time, which might be influenced by time‐on‐task (Maffei and Angrilli 2018). Therefore, the extent to which alpha increases are related to artifact‐ or higher‐level time‐on‐task factors (e.g., mind wandering) remains to be determined by further work. Relatedly, although ocular artifacts were identified and removed through ICA, the current EEG montage comprises a limited number of six channels. This may have affected the robustness of ICA decomposition and corresponding artifact correction (although see Tables S4 and S10).

## 5 Conclusion

This study shows that alpha power increases spontaneously over time during an EEG‐NF session without instructions and engagement in self‐regulation. These increases are similar to those targeted and observed in genuine and sham EEG‐NF contexts, supporting the view that spontaneous EEG dynamics and non‐specific contextual factors can influence the EEG features targeted by EEG‐NF. We suggest that future research should incorporate appropriate control conditions and explicitly investigate the mechanisms driving EEG modulation to better characterize and target EEG‐NF effects.

## Author Contributions

**Jacob Maaz:** conceptualization, investigation, data curation, formal analysis, visualization, resources, writing – review and editing, writing – original draft, project administration, methodology. **Véronique Paban:** conceptualization, methodology, project administration, resources, supervision, writing – review and editing. **Laurent Waroquier:** conceptualization, methodology, supervision, writing – review and editing. **Arnaud Rey:** conceptualization, methodology, project administration, resources, writing – review and editing, supervision.

## Funding

J.M. was supported by a doctoral fellowship of the French Ministry of Higher Education, Research, and Innovation. V.P. was supported by the Fond de dotation JANSSEN HORIZON (grant numbers CNRS SPV/MB/214535). This research was supported by the Convergence Institute ILCB (ANR‐16‐CONV‐0002), the NeuroMarseille Institute and the NeuroSchool PhD program, the Centre for Research in Education Ampiric, and the HEBBIAN ANR project (#ANR‐23‐CE28‐0008). For the purpose of Open Access, a CC‐BY 4.0 public copyright license has been applied by the authors to the present document and will be applied to all subsequent versions up to the Author Accepted Manuscript arising from this submission.

## Conflicts of Interest

The authors declare no conflicts of interest.

## Supporting information


**Table S1:** Participants' subjective feeling of control on the circle variations (only the group with online alpha‐driven variations).
**Table S2:** Checklist guidelines for time‐frequency analyses (adapted from Keil et al. 2022).
**Table S3:** Checklist guidelines for spectral analyses (adapted from Keil et al. 2022).
**Table S4:** Number of Independent Components removed from the data of each participant.
**Table S5:** Custom‐coded contrast matrix assigned to the Condition predictor of each model.
**Table S6:** Custom‐coded contrast matrix assigned to the Source predictor of each model.
**Table S7:** Estimates of the trial repetition effect within the control condition.
**Table S8:** Estimates from statistical models computed with Equation (1).
**Table S9:** Estimates from models considering the ‘Trial’ predictor (integers of 1–32) throughout the entire task.
**Table S10:** Estimates from models computed with Equation (1) on EEG data without ICA‐based artifact correction.
**Figure S1:** Averaged power spectra for each trial. Power spectra depending on the trial number (from clearer to darker colors: 1:8) and on electrode position (A: Fz; B: Cz; C: Pz). Each spectrum has been obtained by averaging spectral power across participants and conditions with a frequency resolution of ~0.305 Hz. On each panel, vertical dashed lines represent the boundaries each of the frequency bands considered as dependent variables, that is, theta (4–8 Hz), alpha (8–12 Hz), SMR (12–15 Hz) and beta (15–30 Hz). Consistently with the statistical results (see Table S8), substantial increases in power are solely observed within the alpha range for each electrode.
**Figure S2:** Averaged power spectra depending on the presence of continuous circle size modification and its corresponding frequency rate. Power spectra depending on the condition (cream: control—no circle modification; red: 1 Hz; violet: 5 Hz; dark blue: 10 Hz) and on electrode position (A: Fz; B: Cz; C: Pz). Each spectrum has been obtained by averaging spectral power across participants and trials with a frequency resolution of ~0.305 Hz. On each panel, vertical dashed lines represent each of the feedback update frequencies.

## Data Availability

All materials, data, and analysis codes are available via the Open Science Framework: https://osf.io/yp8fw.

## References

[psyp70285-bib-0001] Agnoli, S. , M. Zanon , S. Mastria , A. Avenanti , and G. E. Corazza . 2018. “Enhancing Creative Cognition With a Rapid Right‐Parietal Neurofeedback Procedure.” Neuropsychologia 118: 99–106. 10.1016/j.neuropsychologia.2018.02.015. 29454010

[psyp70285-bib-0002] Alkoby, O. , A. Abu‐Rmileh , O. Shriki , and D. Todder . 2018. “Can we Predict Who Will Respond to Neurofeedback? A Review of the Inefficacy Problem and Existing Predictors for Successful EEG Neurofeedback Learning.” Neuroscience 378: 155–164. 10.1016/j.neuroscience.2016.12.050. 28069531

[psyp70285-bib-0003] Arnau, S. , T. Brümmer , N. Liegel , and E. Wascher . 2021. “Inverse Effects of Time‐On‐Task in Task‐Related and Task‐Unrelated Theta Activity.” Psychophysiology 58, no. 6: e13805. 10.1111/psyp.13805. 33682172

[psyp70285-bib-0004] Arnau, S. , C. Löffler , J. Rummel , D. Hagemann , E. Wascher , and A.‐L. Schubert . 2020. “Inter‐Trial Alpha Power Indicates Mind Wandering.” Psychophysiology 57, no. 6: e13581. 10.1111/psyp.13581. 32277853

[psyp70285-bib-0005] Arns, M. , J.‐M. Batail , S. Bioulac , et al. 2017. “Neurofeedback: One of Today's Techniques in Psychiatry?” L'Encephale 43, no. 2: 135–145. 10.1016/j.encep.2016.11.003. 28041692

[psyp70285-bib-0006] Bagherzadeh, Y. , D. Baldauf , D. Pantazis , and R. Desimone . 2020. “Alpha Synchrony and the Neurofeedback Control of Spatial Attention.” Neuron 105, no. 3: 577–587. 10.1016/j.neuron.2019.11.001. 31812515

[psyp70285-bib-0007] Barr, D. J. , R. Levy , C. Scheepers , and H. J. Tily . 2013. “Random Effects Structure for Confirmatory Hypothesis Testing: Keep It Maximal.” Journal of Memory and Language 68, no. 3: 255–278. 10.1016/j.jml.2012.11.001. PMC3881361 24403724

[psyp70285-bib-0008] Belinskaia, A. , N. Smetanin , M. Lebedev , and A. Ossadtchi . 2020. “Short‐Delay Neurofeedback Facilitates Training of the Parietal Alpha Rhythm.” Journal of Neural Engineering 17, no. 6: 066012. 10.1088/1741-2552/abc8d7. 33166941

[psyp70285-bib-0009] Benwell, C. S. Y. , R. E. London , C. F. Tagliabue , et al. 2019. “Frequency and Power of Human Alpha Oscillations Drift Systematically With Time‐On‐Task.” NeuroImage 192: 101–114. 10.1016/j.neuroimage.2019.02.067. 30844505 PMC6503153

[psyp70285-bib-0010] Berger, A. M. , and E. J. Davelaar . 2018. “Frontal Alpha Oscillations and Attentional Control: A Virtual Reality Neurofeedback Study.” Neuroscience 378: 189–197. 10.1016/j.neuroscience.2017.06.007. 28642166

[psyp70285-bib-0011] Bing‐Canar, H. , J. Pizzuto , and R. J. Compton . 2016. “Mindfulness‐Of‐Breathing Exercise Modulates EEG Alpha Activity During Cognitive Performance.” Psychophysiology 53, no. 9: 1366–1376. 10.1111/psyp.12678. 27245493

[psyp70285-bib-0012] Boe, S. , A. Gionfriddo , S. Kraeutner , A. Tremblay , G. Little , and T. Bardouille . 2014. “Laterality of Brain Activity During Motor Imagery Is Modulated by the Provision of Source Level Neurofeedback.” NeuroImage 101: 159–167. 10.1016/j.neuroimage.2014.06.066. 24999037

[psyp70285-bib-0013] Bürkner, P.‐C. 2017. “Brms: An R Package for Bayesian Multilevel Models Using Stan.” Journal of Statistical Software 80: 1–28. 10.18637/jss.v080.i01.

[psyp70285-bib-0014] Chiasson, P. , M. R. Boylan , M. Elhamiasl , et al. 2023. “Effects of Neurofeedback Training on Performance in Laboratory Tasks: A Systematic Review.” International Journal of Psychophysiology 189: 42–56. 10.1016/j.ijpsycho.2023.04.005. 37148977

[psyp70285-bib-0015] Chikhi, S. , N. Matton , M. Sanna , and S. Blanchet . 2023. “Mental Strategies and Resting State EEG: Effect on High Alpha Amplitude Modulation by Neurofeedback in Healthy Young Adults.” Biological Psychology 178: 108521. 10.1016/j.biopsycho.2023.108521. 36801435

[psyp70285-bib-0016] Chikhi, S. , N. Matton , M. Sanna , and S. Blanchet . 2024. “Effects of One Session of Theta or High Alpha Neurofeedback on EEG Activity and Working Memory.” Cognitive, Affective, & Behavioral Neuroscience 24, no. 6: 1065–1083. 10.3758/s13415-024-01218-4. 39322825

[psyp70285-bib-0017] Compton, R. J. , D. Gearinger , and H. Wild . 2019. “The Wandering Mind Oscillates: EEG Alpha Power Is Enhanced During Moments of Mind‐Wandering.” Cognitive, Affective, & Behavioral Neuroscience 19, no. 5: 1184–1191. 10.3758/s13415-019-00745-9. 31502206

[psyp70285-bib-0018] Compton, R. J. , D. Shudrenko , K. Mann , E. Turdukulov , E. Ng , and L. Miller . 2024. “Effects of Task Context on EEG Correlates of Mind‐Wandering.” Cognitive, Affective, & Behavioral Neuroscience 24, no. 1: 72–86. 10.3758/s13415-023-01138-9. PMC10827903 38030911

[psyp70285-bib-0019] Dekker, M. K. J. , M. M. Sitskoorn , A. J. M. Denissen , and G. J. M. van Boxtel . 2014. “The Time‐Course of Alpha Neurofeedback Training Effects in Healthy Participants.” Biological Psychology 95: 70–73. 10.1016/j.biopsycho.2013.11.014. 24321361

[psyp70285-bib-0020] Delorme, A. , and S. Makeig . 2004. “EEGLAB: An Open Source Toolbox for Analysis of Single‐Trial EEG Dynamics Including Independent Component Analysis.” Journal of Neuroscience Methods 134, no. 1: 9–21. 10.1016/j.jneumeth.2003.10.009. 15102499

[psyp70285-bib-0021] Delorme, A. , T. Sejnowski , and S. Makeig . 2007. “Enhanced Detection of Artifacts in EEG Data Using Higher‐Order Statistics and Independent Component Analysis.” NeuroImage 34, no. 4: 1443–1449. 10.1016/j.neuroimage.2006.11.004. 17188898 PMC2895624

[psyp70285-bib-0022] Dempster, T. , and D. Vernon . 2009. “Identifying Indices of Learning for Alpha Neurofeedback Training.” Applied Psychophysiology and Biofeedback 34, no. 4: 309–318. 10.1007/s10484-009-9112-3. 19760142

[psyp70285-bib-0023] Dessy, E. , O. Mairesse , M. van Puyvelde , A. Cortoos , X. Neyt , and N. Pattyn . 2020. “Train Your Brain? Can we Really Selectively Train Specific EEG Frequencies With Neurofeedback Training.” Frontiers in Human Neuroscience 14: 22. 10.3389/fnhum.2020.00022. 32210777 PMC7077336

[psyp70285-bib-0024] Dessy, E. , M. Van Puyvelde , O. Mairesse , X. Neyt , and N. Pattyn . 2018. “Cognitive Performance Enhancement: Do Biofeedback and Neurofeedback Work?” Journal of Cognitive Enhancement 2, no. 1: 12–42. 10.1007/s41465-017-0039-y.

[psyp70285-bib-0025] Dienes, Z. 2014. “Using Bayes to Get the Most Out of Non‐Significant Results.” Frontiers in Psychology 5: 781. 10.3389/fpsyg.2014.00781. 25120503 PMC4114196

[psyp70285-bib-0026] Dienes, Z. , and N. Mclatchie . 2018. “Four Reasons to Prefer Bayesian Analyses Over Significance Testing.” Psychonomic Bulletin & Review 25, no. 1: 207–218. 10.3758/s13423-017-1266-z. 28353065 PMC5862925

[psyp70285-bib-0027] Egner, T. , and J. H. Gruzelier . 2004. “The Temporal Dynamics of Electroencephalographic Responses to Alpha/Theta Neurofeedback Training in Healthy Subjects.” Journal of Neurotherapy 8, no. 1: 43–57. 10.1300/J184v08n01_04.

[psyp70285-bib-0028] Egner, T. , T. F. Zech , and J. H. Gruzelier . 2004. “The Effects of Neurofeedback Training on the Spectral Topography of the Electroencephalogram.” Clinical Neurophysiology 115, no. 11: 2452–2460. 10.1016/j.clinph.2004.05.033. 15465432

[psyp70285-bib-0029] Enriquez‐Geppert, S. , R. J. Huster , C. Figge , and C. S. Herrmann . 2014. “Self‐Regulation of Frontal‐Midline Theta Facilitates Memory Updating and Mental Set Shifting.” Frontiers in Behavioral Neuroscience 8: 420. 10.3389/fnbeh.2014.00420. 25538585 PMC4257088

[psyp70285-bib-0030] Enriquez‐Geppert, S. , R. J. Huster , and C. S. Herrmann . 2017. “EEG‐Neurofeedback as a Tool to Modulate Cognition and Behavior: A Review Tutorial.” Frontiers in Human Neuroscience 11: 51. 10.3389/fnhum.2017.00051. 28275344 PMC5319996

[psyp70285-bib-0031] Enriquez‐Geppert, S. , R. J. Huster , R. Scharfenort , Z. N. Mokom , J. Zimmermann , and C. S. Herrmann . 2014. “Modulation of Frontal‐Midline Theta by Neurofeedback.” Biological Psychology 95: 59–69. 10.1016/j.biopsycho.2013.02.019. 23499994

[psyp70285-bib-0032] Enriquez‐Geppert, S. , D. Smit , M. G. Pimenta , and M. Arns . 2019. “Neurofeedback as a Treatment Intervention in ADHD: Current Evidence and Practice.” Current Psychiatry Reports 21, no. 6: 46. 10.1007/s11920-019-1021-4. 31139966 PMC6538574

[psyp70285-bib-0033] Escolano, C. , M. Navarro‐Gil , J. Garcia‐Campayo , and J. Minguez . 2014. “The Effects of a Single Session of Upper Alpha Neurofeedback for Cognitive Enhancement: A Sham‐Controlled Study.” Applied Psychophysiology and Biofeedback 39, no. 3: 227–236. 10.1007/s10484-014-9262-9. 25267413

[psyp70285-bib-0034] Fingelkurts, A. A. , and A. A. Fingelkurts . 2006. “Timing in Cognition and EEG Brain Dynamics: Discreteness Versus Continuity.” Cognitive Processing 7, no. 3: 135–162. 10.1007/s10339-006-0035-0. 16832687

[psyp70285-bib-0035] Fovet, T. , R. Jardri , and J.‐A. Micoulaud‐Franchi . 2016. “Le neurofeedback en psychiatrie: Les outils d'imagerie cérébrale et de neuropsychologie au service de la thérapeutique.” L'Information Psychiatrique 92, no. 4: 285–293.

[psyp70285-bib-0036] Gaume, A. , A. Vialatte , A. Mora‐Sánchez , C. Ramdani , and F. B. Vialatte . 2016. “A Psychoengineering Paradigm for the Neurocognitive Mechanisms of Biofeedback and Neurofeedback.” Neuroscience & Biobehavioral Reviews 68: 891–910. 10.1016/j.neubiorev.2016.06.012. 27339691

[psyp70285-bib-0037] Gelman, A. , J. B. Carlin , H. S. Stern , D. B. Dunson , A. Vehtari , and D. B. Rubin . 2014. Bayesian Data Analysis. Third ed. Chapman and Hall/CRC.

[psyp70285-bib-0038] Gelman, A. , J. Hill , and M. Yajima . 2012. “Why we (Usually) Don't Have to Worry About Multiple Comparisons.” Journal of Research on Educational Effectiveness 5, no. 2: 189–211. 10.1080/19345747.2011.618213.

[psyp70285-bib-0039] Gonçalves, Ó. F. , S. Carvalho , A. J. Mendes , J. Leite , and P. S. Boggio . 2018. “Neuromodulating Attention and Mind‐Wandering Processes With a Single Session Real Time EEG.” Applied Psychophysiology and Biofeedback 43, no. 2: 143–151. 10.1007/s10484-018-9394-4. 29797155

[psyp70285-bib-0040] Gouraud, J. , A. Delorme , and B. Berberian . 2018. “Influence of Automation on Mind Wandering Frequency in Sustained Attention.” Consciousness and Cognition 66: 54–64. 10.1016/j.concog.2018.09.012. 30396034

[psyp70285-bib-0041] Grosselin, F. , A. Breton , L. Yahia‐Cherif , et al. 2021. “Alpha Activity Neuromodulation Induced by Individual Alpha‐Based Neurofeedback Learning in Ecological Context: A Double‐Blind Randomized Study.” Scientific Reports 11, no. 1: 18489. 10.1038/s41598-021-96893-5. 34531416 PMC8445968

[psyp70285-bib-0042] Gruzelier, J. H. 2014a. “EEG‐Neurofeedback for Optimising Performance. I: A Review of Cognitive and Affective Outcome in Healthy Participants.” Neuroscience & Biobehavioral Reviews 44: 124–141. 10.1016/j.neubiorev.2013.09.015. 24125857

[psyp70285-bib-0043] Gruzelier, J. H. 2014b. “EEG‐Neurofeedback for Optimising Performance. III: A Review of Methodological and Theoretical Considerations.” Neuroscience & Biobehavioral Reviews 44: 159–182. 10.1016/j.neubiorev.2014.03.015. 24690579

[psyp70285-bib-0044] Hammond, D. C. 2005. “Neurofeedback Treatment of Depression and Anxiety.” Journal of Adult Development 12, no. 2: 131–137. 10.1007/s10804-005-7029-5.

[psyp70285-bib-0045] Hanslmayr, S. , J. Gross , W. Klimesch , and K. L. Shapiro . 2011. “The Role of Alpha Oscillations in Temporal Attention.” Brain Research Reviews 67, no. 1: 331–343. 10.1016/j.brainresrev.2011.04.002. 21592583

[psyp70285-bib-0046] Hardt, J. V. , and J. Kamiya . 1978. “Anxiety Change Through Electroencephalographic Alpha Feedback Seen Only in High Anxiety Subjects.” Science 201, no. 4350: 79–81. 10.1126/science.663641. 663641

[psyp70285-bib-0047] Hasslinger, J. , M. Meregalli , and S. Bölte . 2022. “How Standardized Are “Standard Protocols”? Variations in Protocol and Performance Evaluation for Slow Cortical Potential Neurofeedback: A Systematic Review.” Frontiers in Human Neuroscience 16: 887504. 10.3389/fnhum.2022.887504. 36118975 PMC9478392

[psyp70285-bib-0048] Hsueh, J.‐J. , T.‐S. Chen , J.‐J. Chen , and F.‐Z. Shaw . 2016. “Neurofeedback Training of EEG Alpha Rhythm Enhances Episodic and Working Memory.” Human Brain Mapping 37, no. 7: 2662–2675. 10.1002/hbm.23201. 27038114 PMC6867560

[psyp70285-bib-0049] Jeffreys, H. 1939. Theory of Probability. Clarendon Press.

[psyp70285-bib-0050] Jensen, M. , J. C. G. Alanis , E. Hüttenrauch , et al. 2023. “Does It Matter What Is Trained? A Randomized Controlled Trial Evaluating the Specificity of Alpha/delta Ratio Neurofeedback in Reducing Tinnitus Symptoms.” Brain Communications 5, no. 4: fcad185. 10.1093/braincomms/fcad185. 37680692 PMC10481778

[psyp70285-bib-0051] Jiang, H. , J. Stieger , M. J. Kreitzer , S. Engel , and B. He . 2021. “Frontolimbic Alpha Activity Tracks Intentional Rest BCI Control Improvement Through Mindfulness Meditation.” Scientific Reports 11, no. 1: 6818. 10.1038/s41598-021-86215-0. 33767254 PMC7994299

[psyp70285-bib-0052] Kadosh, K. C. , and G. Staunton . 2019. “A Systematic Review of the Psychological Factors That Influence Neurofeedback Learning Outcomes.” NeuroImage 185: 545–555. 10.1016/j.neuroimage.2018.10.021. 30315905

[psyp70285-bib-0053] Kalokairinou, L. , L. S. Sullivan , and A. Wexler . 2022. “Neurofeedback as Placebo: A Case of Unintentional Deception?” Journal of Medical Ethics 48, no. 12: 1037–1042. 10.1136/medethics-2021-107435. 34521768 PMC9205641

[psyp70285-bib-0054] Keil, A. , E. M. Bernat , M. X. Cohen , et al. 2022. “Recommendations and Publication Guidelines for Studies Using Frequency Domain and Time‐Frequency Domain Analyses of Neural Time Series.” Psychophysiology 59, no. 5: e14052. 10.1111/psyp.14052. 35398913 PMC9717489

[psyp70285-bib-0055] Kleiner, M. , D. Brainard , and D. Pelli . 2007. What's New in Psychtoolbox‐3? 30th European Conference on Visual Perception. https://pure.mpg.de/rest/items/item_1790332/component/file_3136265/content.

[psyp70285-bib-0056] Klimesch, W. 1999. “EEG Alpha and Theta Oscillations Reflect Cognitive and Memory Performance: A Review and Analysis.” Brain Research Reviews 29, no. 2: 169–195. 10.1016/S0165-0173(98)00056-3. 10209231

[psyp70285-bib-0057] Klimesch, W. 2012. “Alpha‐Band Oscillations, Attention, and Controlled Access to Stored Information.” Trends in Cognitive Sciences 16, no. 12: 606–617. 10.1016/j.tics.2012.10.007. 23141428 PMC3507158

[psyp70285-bib-0058] Klimesch, W. , P. Sauseng , and S. Hanslmayr . 2007. “EEG Alpha Oscillations: The Inhibition–Timing Hypothesis.” Brain Research Reviews 53, no. 1: 63–88. 10.1016/j.brainresrev.2006.06.003. 16887192

[psyp70285-bib-0059] Kluetsch, R. C. , T. Ros , J. Théberge , et al. 2014. “Plastic Modulation of PTSD Resting‐State Networks and Subjective Wellbeing by EEG Neurofeedback.” Acta Psychiatrica Scandinavica 130, no. 2: 123–136. 10.1111/acps.12229. 24266644 PMC4442612

[psyp70285-bib-0060] Kober, S. E. , M. Witte , M. Stangl , A. Väljamäe , C. Neuper , and G. Wood . 2015. “Shutting Down Sensorimotor Interference Unblocks the Networks for Stimulus Processing: An SMR Neurofeedback Training Study.” Clinical Neurophysiology 126, no. 1: 82–95. 10.1016/j.clinph.2014.03.031. 24794517

[psyp70285-bib-0061] Kopčanová, M. , G. Thut , C. S. Y. Benwell , and C. Keitel . 2025. “Characterising Time‐On‐Task Effects on Oscillatory and Aperiodic EEG Components and Their Co‐Variation With Visual Task Performance.” Imaging Neuroscience 3: 00566. 10.1162/imag_a_00566. PMC12319923 40800988

[psyp70285-bib-0062] Lee, Y.‐J. , H.‐G. Kim , E.‐J. Cheon , et al. 2019. “The Analysis of Electroencephalography Changes Before and After a Single Neurofeedback Alpha/Theta Training Session in University Students.” Applied Psychophysiology and Biofeedback 44, no. 3: 173–184. 10.1007/s10484-019-09432-4. 30903394

[psyp70285-bib-0063] Maaz, J. , V. Paban , L. Waroquier , and A. Rey . 2025. “Spontaneous Modulation of Standard EEG Frequency Bands During a Neurofeedback‐Like Task.” Psychophysiology 62, no. 10: e70163. 10.1111/psyp.70163. 41045022 PMC12495449

[psyp70285-bib-0064] Maffei, A. , and A. Angrilli . 2018. “Spontaneous Eye Blink Rate: An Index of Dopaminergic Component of Sustained Attention and Fatigue.” International Journal of Psychophysiology 123: 58–63. 10.1016/j.ijpsycho.2017.11.009. 29133149

[psyp70285-bib-0065] Magosso, E. , F. De Crescenzio , G. Ricci , S. Piastra , and M. Ursino . 2019. “EEG Alpha Power Is Modulated by Attentional Changes During Cognitive Tasks and Virtual Reality Immersion.” Computational Intelligence and Neuroscience 2019, no. 1: 7051079. 10.1155/2019/7051079. 31341468 PMC6614966

[psyp70285-bib-0066] Maszczyk, A. , P. Dobrakowski , M. Nitychoruk , M. Żak , M. Kowalczyk , and M. Toborek . 2020. “The Effect of Neurofeedback Training on the Visual Processing Efficiency in Judo Athletes.” Journal of Human Kinetics 71, no. 1: 219–227. 10.2478/hukin-2019-0097. 32148586 PMC7052722

[psyp70285-bib-0067] Michel, C. M. , and T. Koenig . 2018. “EEG Microstates as a Tool for Studying the Temporal Dynamics of Whole‐Brain Neuronal Networks: A Review.” NeuroImage 180: 577–593. 10.1016/j.neuroimage.2017.11.062. 29196270

[psyp70285-bib-0068] Micoulaud Franchi, J.‐A. , C. Jeunet , and F. Lotte . 2020. “Neurofeedback: A Challenge for Integrative Clinical Neurophysiological Studies.” Neurophysiologie Clinique 50, no. 1: 1–3. 10.1016/j.neucli.2020.01.001. 32007382

[psyp70285-bib-0069] Micoulaud‐Franchi, J.‐A. , J.‐M. Batail , T. Fovet , et al. 2019. “Towards a Pragmatic Approach to a Psychophysiological Unit of Analysis for Mental and Brain Disorders: An EEG‐Copeia for Neurofeedback.” Applied Psychophysiology and Biofeedback 44, no. 3: 151–172. 10.1007/s10484-019-09440-4. 31098793

[psyp70285-bib-0070] Micoulaud‐Franchi, J.‐A. , and T. Fovet . 2016. “Neurofeedback: Time Needed for a Promising Non‐Pharmacological Therapeutic Method.” Lancet Psychiatry 3, no. 9: e16. 10.1016/S2215-0366(16)30189-4. 27568274

[psyp70285-bib-0071] Micoulaud‐Franchi, J.‐A. , and T. Fovet . 2018. “A Framework for Disentangling the Hyperbolic Truth of Neurofeedback: Comment on Thibault and Raz (2017).” American Psychologist 73, no. 7: 933–935. 10.1037/amp0000340. 30284893

[psyp70285-bib-0072] Micoulaud‐Franchi, J.‐A. , A. McGonigal , R. Lopez , C. Daudet , I. Kotwas , and F. Bartolomei . 2015. “Electroencephalographic Neurofeedback: Level of Evidence in Mental and Brain Disorders and Suggestions for Good Clinical Practice.” Neurophysiologie Clinique/Clinical Neurophysiology 45, no. 6: 423–433. 10.1016/j.neucli.2015.10.077. 26553293

[psyp70285-bib-0073] Mirifar, A. , A. Keil , and F. Ehrlenspiel . 2022. “Neurofeedback and Neural Self‐Regulation: A New Perspective Based on Allostasis.” Reviews in the Neurosciences 33, no. 6: 607–629. 10.1515/revneuro-2021-0133. 35122709 PMC9381001

[psyp70285-bib-0074] Muñoz‐Moldes, S. , and A. Cleeremans . 2020. “Delineating Implicit and Explicit Processes in Neurofeedback Learning.” Neuroscience & Biobehavioral Reviews 118: 681–688. 10.1016/j.neubiorev.2020.09.003. 32918947 PMC7758707

[psyp70285-bib-0075] Naas, A. , J. Rodrigues , J.‐P. Knirsch , and A. Sonderegger . 2019. “Neurofeedback Training With a Low‐Priced EEG Device Leads to Faster Alpha Enhancement but Shows no Effect on Cognitive Performance: A Single‐Blind, Sham‐Feedback Study.” PLoS One 14, no. 9: e0211668. 10.1371/journal.pone.0211668. 31483789 PMC6726238

[psyp70285-bib-0076] Neurofeedback Collaborative Group . 2021. “Double‐Blind Placebo‐Controlled Randomized Clinical Trial of Neurofeedback for Attention‐Deficit/Hyperactivity Disorder With 13‐Month Follow‐Up.” Journal of the American Academy of Child & Adolescent Psychiatry 60, no. 7: 841–855. 10.1016/j.jaac.2020.07.906. 32853703 PMC7904968

[psyp70285-bib-0077] Neurofeedback Collaborative Group . 2023. “Neurofeedback for Attention‐Deficit/Hyperactivity Disorder: 25‐Month Follow‐Up of Double‐Blind Randomized Controlled Trial.” Journal of the American Academy of Child & Adolescent Psychiatry 62, no. 4: 435–446. 10.1016/j.jaac.2022.07.862. 36521694 PMC10065891

[psyp70285-bib-0078] Nowlis, D. P. , and J. Kamiya . 1970. “The Control of Electroencephalographic Alpha Rhythms Through Auditory Feedback and the Associated Mental Activity.” Psychophysiology 6, no. 4: 476–484. 10.1111/j.1469-8986.1970.tb01756.x. 5418812

[psyp70285-bib-0079] Peeters, F. , M. Oehlen , J. Ronner , J. van Os , and R. Lousberg . 2014. “Neurofeedback as a Treatment for Major Depressive Disorder – A Pilot Study.” PLoS One 9, no. 3: e91837. 10.1371/journal.pone.0091837. 24642756 PMC3958393

[psyp70285-bib-0080] Pigott, H. E. , R. Cannon , and M. Trullinger . 2018. “The Fallacy of Sham‐Controlled Neurofeedback Trials: A Reply to Thibault and Colleagues (2018).” Journal of Attention Disorders 25, no. 3: 448–457. 10.1177/1087054718790802. 30078340 PMC7783691

[psyp70285-bib-0081] Pigott, H. E. , M. Trullinger , H. Harbin , J. Cammack , F. Harbin , and R. Cannon . 2017. “Confusion Regarding Operant Conditioning of the EEG.” Lancet Psychiatry 4, no. 12: 897. 10.1016/S2215-0366(17)30436-4. 29179924

[psyp70285-bib-0082] R Core Team . 2024. R: The R Project for Statistical Computing [Computer Software]. https://www.r‐project.org/.

[psyp70285-bib-0083] Ramot, M. , and A. Martin . 2022. “Closed‐Loop Neuromodulation for Studying Spontaneous Activity and Causality.” Trends in Cognitive Sciences 26, no. 4: 290–299. 10.1016/j.tics.2022.01.008. 35210175 PMC9396631

[psyp70285-bib-0084] Rogala, J. , K. Jurewicz , K. Paluch , E. Kublik , R. Cetnarski , and A. Wróbel . 2016. “The Do's and Don'ts of Neurofeedback Training: A Review of the Controlled Studies Using Healthy Adults.” Frontiers in Human Neuroscience 10: 301. 10.3389/fnhum.2016.00301. 27378892 PMC4911408

[psyp70285-bib-0085] Ros, T. , S. Enriquez‐Geppert , V. Zotev , et al. 2020. “Consensus on the Reporting and Experimental Design of Clinical and Cognitive‐Behavioural Neurofeedback Studies (CRED‐Nf Checklist).” Brain 143, no. 6: 1674–1685. 10.1093/brain/awaa009. 32176800 PMC7296848

[psyp70285-bib-0086] Rozengurt, R. , A. Barnea , S. Uchida , and D. A. Levy . 2016. “Theta EEG Neurofeedback Benefits Early Consolidation of Motor Sequence Learning.” Psychophysiology 53, no. 7: 965–973. 10.1111/psyp.12656. 27080752

[psyp70285-bib-0087] Salari, N. , C. Büchel , and M. Rose . 2014. “Neurofeedback Training of Gamma Band Oscillations Improves Perceptual Processing.” Experimental Brain Research 232, no. 10: 3353–3361. 10.1007/s00221-014-4023-9. 24992898

[psyp70285-bib-0088] Sauseng, P. , W. Klimesch , W. Stadler , et al. 2005. “A Shift of Visual Spatial Attention Is Selectively Associated With Human EEG Alpha Activity.” European Journal of Neuroscience 22, no. 11: 2917–2926. 10.1111/j.1460-9568.2005.04482.x. 16324126

[psyp70285-bib-0089] Schabus, M. 2017. “Reply: On Assessing Neurofeedback Effects: Should Double‐Blind Replace Neurophysiological Mechanisms?” Brain: A Journal of Neurology 140, no. 10: e64. 10.1093/brain/awx212. 28969379 PMC5695662

[psyp70285-bib-0090] Schabus, M. 2018. “Reply: Noisy but Not Placebo: Defining Metrics for Effects of Neurofeedback.” Brain 141, no. 5: e41. 10.1093/brain/awy061. 29547955 PMC5917747

[psyp70285-bib-0091] Schabus, M. , H. Griessenberger , M.‐T. Gnjezda , D. P. J. Heib , M. Wislowska , and K. Hoedlmoser . 2017. “Better Than Sham? A Double‐Blind Placebo‐Controlled Neurofeedback Study in Primary Insomnia.” Brain 140, no. 4: 1041–1052. 10.1093/brain/awx011. 28335000 PMC5382955

[psyp70285-bib-0092] Schad, D. J. , M. Betancourt , and S. Vasishth . 2021. “Toward a Principled Bayesian Workflow in Cognitive Science.” Psychological Methods 26, no. 1: 103–126. 10.1037/met0000275. 32551748

[psyp70285-bib-0093] Schad, D. J. , B. Nicenboim , P.‐C. Bürkner , M. Betancourt , and S. Vasishth . 2023. “Workflow Techniques for the Robust Use of Bayes Factors.” Psychological Methods 28, no. 6: 1404–1426. 10.1037/met0000472. 35266787

[psyp70285-bib-0094] Schad, D. J. , S. Vasishth , S. Hohenstein , and R. Kliegl . 2020. “How to Capitalize on a Priori Contrasts in Linear (Mixed) Models: A Tutorial.” Journal of Memory and Language 110: 104038. 10.1016/j.jml.2019.104038.

[psyp70285-bib-0095] Schönbrodt, F. D. , and E.‐J. Wagenmakers . 2018. “Bayes Factor Design Analysis: Planning for Compelling Evidence.” Psychonomic Bulletin & Review 25, no. 1: 128–142. 10.3758/s13423-017-1230-y. 28251595

[psyp70285-bib-0096] Schönenberg, M. , A.‐L. Weingärtner , K. Weimer , and J. Scheeff . 2021. “Believing Is Achieving—On the Role of Treatment Expectation in Neurofeedback Applications.” Progress in Neuro‐Psychopharmacology and Biological Psychiatry 105: 110129. 10.1016/j.pnpbp.2020.110129. 33031860

[psyp70285-bib-0097] Schönenberg, M. , E. Wiedemann , A. Schneidt , et al. 2017. “Neurofeedback, Sham Neurofeedback, and Cognitive‐Behavioural Group Therapy in Adults With Attention‐Deficit Hyperactivity Disorder: A Triple‐Blind, Randomised, Controlled Trial.” Lancet Psychiatry 4, no. 9: 673–684. 10.1016/S2215-0366(17)30291-2. 28803030

[psyp70285-bib-0098] Sitaram, R. , T. Ros , L. Stoeckel , et al. 2017. “Closed‐Loop Brain Training: The Science of Neurofeedback.” Nature Reviews Neuroscience 18, no. 2: 2. 10.1038/nrn.2016.164. 28003656

[psyp70285-bib-0099] Sorger, B. , F. Scharnowski , D. E. J. Linden , M. Hampson , and K. D. Young . 2019. “Control Freaks: Towards Optimal Selection of Control Conditions for fMRI Neurofeedback Studies.” NeuroImage 186: 256–265. 10.1016/j.neuroimage.2018.11.004. 30423429 PMC6338498

[psyp70285-bib-0100] Spaak, E. , F. P. de Lange , and O. Jensen . 2014. “Local Entrainment of Alpha Oscillations by Visual Stimuli Causes Cyclic Modulation of Perception.” Journal of Neuroscience 34, no. 10: 3536–3544. 10.1523/JNEUROSCI.4385-13.2014. 24599454 PMC6608988

[psyp70285-bib-0101] Stan Development Team . 2024. RStan: The R Interface to Stan [Computer Software]. https://mc‐stan.org/.

[psyp70285-bib-0102] Stokes, D. A. , and M. S. Lappin . 2010. “Neurofeedback and Biofeedback With 37 Migraineurs: A Clinical Outcome Study.” Behavioral and Brain Functions 6, no. 1: 9. 10.1186/1744-9081-6-9. 20205867 PMC2826281

[psyp70285-bib-0103] Strehl, U. 2014. “What Learning Theories Can Teach Us in Designing Neurofeedback Treatments.” Frontiers in Human Neuroscience 8: 894. 10.3389/fnhum.2014.00894. 25414659 PMC4222234

[psyp70285-bib-0104] Studer, P. , O. Kratz , H. Gevensleben , et al. 2014. “Slow Cortical Potential and Theta/Beta Neurofeedback Training in Adults: Effects on Attentional Processes and Motor System Excitability.” Frontiers in Human Neuroscience 8: 555. 10.3389/fnhum.2014.00555. 25104932 PMC4109432

[psyp70285-bib-0105] Su, K.‐H. , J.‐J. Hsueh , T. Chen , and F.‐Z. Shaw . 2021. “Validation of Eyes‐Closed Resting Alpha Amplitude Predicting Neurofeedback Learning of Upregulation Alpha Activity.” Scientific Reports 11, no. 1: 19615. 10.1038/s41598-021-99235-7. 34608244 PMC8490456

[psyp70285-bib-0106] Tan, G. , J. Thornby , D. C. Hammond , et al. 2009. “Meta‐Analysis of EEG Biofeedback in Treating Epilepsy.” Clinical EEG and Neuroscience 40, no. 3: 173–179. 10.1177/155005940904000310. 19715180

[psyp70285-bib-0107] Thibault, R. T. , M. Lifshitz , N. Birbaumer , and A. Raz . 2015. “Neurofeedback, Self‐Regulation, and Brain Imaging: Clinical Science and Fad in the Service of Mental Disorders.” Psychotherapy and Psychosomatics 84, no. 4: 193–207. 10.1159/000371714. 26021883

[psyp70285-bib-0108] Thibault, R. T. , M. Lifshitz , and A. Raz . 2016. “The Self‐Regulating Brain and Neurofeedback: Experimental Science and Clinical Promise.” Cortex 74: 247–261. 10.1016/j.cortex.2015.10.024. 26706052

[psyp70285-bib-0109] Thibault, R. T. , and A. Raz . 2017. “The Psychology of Neurofeedback: Clinical Intervention Even if Applied Placebo.” American Psychologist 72, no. 7: 679–688. 10.1037/amp0000118. 29016171

[psyp70285-bib-0110] Thibault, R. T. , and A. Raz . 2018. “A Consensus Framework for Neurofeedback Research (And the Perils of Unfounded Neuroreductionism): Reply to Micoulaud‐Franchi and Fovet (2018).” American Psychologist 73, no. 7: 936–937. 10.1037/amp0000366. 30284894

[psyp70285-bib-0111] van Boxtel, G. J. M. , A. J. M. Denissen , M. Jäger , et al. 2012. “A Novel Self‐Guided Approach to Alpha Activity Training.” International Journal of Psychophysiology 83, no. 3: 282–294. 10.1016/j.ijpsycho.2011.11.004. 22119661

[psyp70285-bib-0112] VanRullen, R. 2016. “Perceptual Cycles.” Trends in Cognitive Sciences 20, no. 10: 723–735. 10.1016/j.tics.2016.07.006. 27567317

[psyp70285-bib-0113] Wan, F. , W. Nan , M. I. Vai , and A. Rosa . 2014. “Resting Alpha Activity Predicts Learning Ability in Alpha Neurofeedback.” Frontiers in Human Neuroscience 8: 500. 10.3389/fnhum.2014.00500. 25071528 PMC4095646

[psyp70285-bib-0114] Wang, C. , R. Rajagovindan , S.‐M. Han , and M. Ding . 2016. “Top‐Down Control of Visual Alpha Oscillations: Sources of Control Signals and Their Mechanisms of Action.” Frontiers in Human Neuroscience 10: 15. 10.3389/fnhum.2016.00015. 26834601 PMC4718979

[psyp70285-bib-0115] Wei, T.‐Y. , D.‐W. Chang , Y.‐D. Liu , et al. 2017. “Portable Wireless Neurofeedback System of EEG Alpha Rhythm Enhances Memory.” Biomedical Engineering Online 16, no. 1: 128. 10.1186/s12938-017-0418-8. 29132359 PMC5684759

[psyp70285-bib-0116] Witte, M. , S. E. Kober , and G. Wood . 2018. “Noisy but Not Placebo: Defining Metrics for Effects of Neurofeedback.” Brain 141, no. 5: e40. 10.1093/brain/awy060. 29547965

[psyp70285-bib-0117] Yeh, W.‐H. , J.‐J. Hsueh , and F.‐Z. Shaw . 2021. “Neurofeedback of Alpha Activity on Memory in Healthy Participants: A Systematic Review and Meta‐Analysis.” Frontiers in Human Neuroscience 14: 562360. 10.3389/fnhum.2020.562360. 33469422 PMC7813983

[psyp70285-bib-0118] Zoefel, B. , R. J. Huster , and C. S. Herrmann . 2011. “Neurofeedback Training of the Upper Alpha Frequency Band in EEG Improves Cognitive Performance.” NeuroImage 54, no. 2: 1427–1431. 10.1016/j.neuroimage.2010.08.078. 20850552

