# Supplementary material for: Spontaneous Modulation of Alpha Power During a Neurofeedback Session Without Instructions
Source: Psychophysiology. 2026 Mar 24;63(3):e70285. doi: 10.1111/psyp.70285 (PMC13011910; doi:10.1111/psyp.70285)
Supplement: Supplementary file 1 — Table S1: Participants' subjective feeling of control on the circle variations (only the group with online alpha‐driven variations). Table S2: Checklist guidelines for time‐frequency analyses (adapted from Keil et al. 2022). Table S3: Checklist guidelines for spectral analyses (adapted from Keil et al. 2022). Table S4: Number of Independent Components removed from the data of each participant. Table S5: Custom‐coded contrast matrix assigned to the Condition predictor of each model. Table S6: Custom‐coded contrast matrix assigned to the Source predictor of each model. Table S7: Estimates of the trial repetition effect within the control condition. Table S8: Estimates from statistical models computed with Equation (1). Table S9: Estimates from models considering the ‘Trial’ predictor (integers of 1–32) throughout the entire task. Table S10: Estimates from models computed with Equation (1) on EEG data without ICA‐based artifact correction. Figure S1: Averaged power spectra for each trial. Power spectra depending on the trial number (from clearer to darker colors: 1:8) and on electrode position (A: Fz; B: Cz; C: Pz). Each spectrum has been obtained by averaging spectral power across participants and conditions with a frequency resolution of ~0.305 Hz. On each panel, vertical dashed lines represent the boundaries each of the frequency bands considered as dependent variables, that is, theta (4–8 Hz), alpha (8–12 Hz), SMR (12–15 Hz) and beta (15–30 Hz). Consistently with the statistical results (see Table S8), substantial increases in power are solely observed within the alpha range for each electrode. Figure S2: Averaged power spectra depending on the presence of continuous circle size modification and its corresponding frequency rate. Power spectra depending on the condition (cream: control—no circle modification; red: 1 Hz; violet: 5 Hz; dark blue: 10 Hz) and on electrode position (A: Fz; B: Cz; C: Pz). Each spectrum has been obtained by averaging spectral power across [file PSYP-63-e70285-s001.docx]

**Supplementary Material for:**

**Spontaneous modulation of alpha power during a neurofeedback session without instructions**

Jacob Maaz,^1,2,3^ Véronique Paban,^1^ Laurent Waroquier^4^ and Arnaud Rey^1,3^

Author affiliations:

1 Aix-Marseille Université, CNRS, CRPN, 13331 Marseille, France

2 Institute Neuro-Marseille, Aix-Marseille Université, France

3 Institute of Language Communication and the Brain, Aix-Marseille Université, France

4 Aix-Marseille Université, PSYCLE, 13621 Aix-en-Provence, France

Correspondence to: Jacob Maaz

Centre for Research in Psychology and Neuroscience (CRPN) – UMR 7077

CNRS – Aix-Marseille Université

3, place Victor Hugo – Case D

13331 Marseille Cedex 3 – France

[jacob.maaz@univ-amu.fr](mailto:jacob.maaz@univ-amu.fr)

**Supplementary Table 1 Participants’ subjective feeling of control on the circle variations (only the group with online alpha-driven variations).**

| **Responses** | **Number of answers** |
| --- | --- |
| 1 – “Not at all” | 17 |
| 2 – “A little” | 9 |
| 3 – “Mildly” | 6 |
| 4 – “A lot” | 0 |
| 5 – “Totally” | 0 |

At the end of the session, participants were asked to answer on a 5-point Likert scale to the question: “During the trials in which the circle was modified, to what extent did you feel you had control over the variations of the circle?”. Most of the participants reported no subjective level of control on the circle variations (*n* = 17), suggesting little explicit knowledge on the link between their EEG activity and the visual feedback. Some also reported subjective feelings of little (*n* = 9) and moderate (*n* = 6) control. Yet, this measure was not implemented in the control group from Maaz et al. (2025), in which the feedback was disconnected from participant EEG activity. Thus, without the possibility for a meaningful between-group comparison, little inference can be sustainably made about these results.

**Supplementary Table 2 Checklist guidelines for time-frequency analyses (adapted from Keil et al., 2022).**

| **#** | **Information to be included in the manuscript** | **Completed?** |
| --- | --- | --- |
| 1 | The specific stage of processing in which time-frequency analysis was applied (e.g., single-trials, after trial averaging, etc.). This clarifies which aspect(s) of oscillatory activity (e.g., spontaneous and/or induced, evoked, etc.) are being observed. If averaged potentials of each trial were subtracted prior to conducting time-frequency analyses on single trials, this step should be stated along with figures depicting the averaged potential in both time and frequency domains. | YES |
| 2 | For authors using Fourier-based time-frequency analyses (spectrograms), the following recommendations are provided for each specific approach: (1) If using spectrograms, or moving-window DFT/FFT analyses, report the specific window size and step size. Additional within-window averaging averaging achieved via algorithms (e.g., Welch periodogram method) should also be reported. (2) If using multitaper analyses, the type of tapering windows used, total number used, their center frequencies, whether any smoothing factors are applied, and the specific algorithms used to form their shapes should be reported. (3) If using complex demodulation, the frequencies examined, and the specific properties of the low-pass filter used (i.e., filter type, order, and cutoff frequency) should be reported. | YES |
| 3 | For authors conducting time-frequency analyses based on time domain filtering methods (i.e., Filter-Hilbert or similar approaches), the software and version number of the Hilbert transform used to identify the phase-shifted version of the empirical signal. In addition, authors should state the specific properties of band-pass filter (i.e., filter types, order, and cutoff frequencies). | Not applicable |
| 4 | If using wavelet-based methods for time-frequency analyses, include the smoothing/ smearing for the minimum and maximum frequency of interest and indicate the maximal temporal and frequency smoothing for a specific wavelet family. In addition, using Morlet wavelets, include the Morlet parameter (*m*) indicating the trade-off between time and frequency smoothing and smoothing values in the time (*σt*) and frequency (*σf*) domains. | Not applicable |
| 5 | As for frequency domain analyses, specify the duration of analytical time segments used, with pre- and post-event onset duration. In addition, include the number of time segments for each condition/group. | YES |
| 6 | Descriptions of any nonlinear transformations and/or baseline adjustment that were used prior to statistical analyses, accompanied by a rationale for these decisions. Specifically, include the duration used as a baseline and the type of algorithm (e.g., division, subtraction, etc.) used for this adjustment. | Not applicable |

**Supplementary Table 3 Checklist guidelines for spectral analyses (adapted from Keil et al., 2022).**

| **#** | **Information to be included in the manuscript** | **Completed?** |
| --- | --- | --- |
| 1 | Specifying the inputs and outputs of all algorithms used in the processing pipeline | YES |
| 2 | A discussion of how oscillatory activity was conceptualised relative to 1/*f* noise and/or other broadband phenomena (underlying model) | YES |
| 3 | A rationale for the choice of measurement of power in a specific frequency band, including how nonperiodic (1/*f*) contributions to the spectrum were addressed | YES |
| 4 | A statement describing the specific type of Fourier- or non-Fourier-based algorithm used for transformation from the time domain to the frequency domain | YES |
| 5 | The exact duration of time segment used for transformation into the frequency domain for each condition of interest. In addition, the total number of segments (e.g., trials per condition) entering an averaged spectrum, along with how data epochs were combined within and across recordings (e.g., overlapping windows) | YES |
| 6 | The type, total number of, overlap between, and duration of any taper window functions, along with their ramp-on and ramp-off duration. If alternative and/or additional steps were taken to address edge artifacts, these should be stated. If applicable, the choice of taper window function should be specified as being guided by computational principles and/or by aiming to replicate current methods (e.g., Hann or Hamming window) | YES |
| 7 | If zero-padding is applied, the number and location of added zeros (e.g., before the time series, after the time series, or both before and after the time series) | Not applicable |
| 8 | All normalisation steps (e.g., by length of time, multiplication of the lower half of the spectrum, or by complex conjugate, etc.) applied to the spectral power or power density calculation | YES |
| 9 | The native frequency resolution of the spectrum (e.g., 1/(epoch duration in seconds)). In addition, the number of frequency bins extracted for a specific band of interest, and the range of these binds (e.g., 7.98 Hz to 11.97 Hz) | YES |
| 10 | Whether analyses were conducted using single trials or the average across trials | YES |
| 11 | How band power was measured from a spectrum | YES |

**Supplementary Table 4 Number of Independent Components removed from the data of each participant.**

| **Participant’s number** | **Number of Independent Components removed** |
| --- | --- |
| 1 | 2 |
| 2 | 2 |
| 3 | 2 |
| 4 | 2 |
| 5 | 2 |
| 6 | 3 |
| 7 | 2 |
| 8 | 1 |
| 9 | 3 |
| 10 | 1 |
| 11 | 2 |
| 12 | 3 |
| 13 | 2 |
| 14 | 3 |
| 15 | 3 |
| 16 | 2 |
| 17 | 2 |
| 18 | 2 |
| 19 | 2 |
| 20 | 2 |
| 21 | 3 |
| 22 | 2 |
| 23 | 2 |
| 24 | 2 |
| 25 | 2 |
| 26 | 2 |
| 27 | 2 |
| 28 | 3 |
| 29 | 3 |
| 30 | 3 |
| 31 | 2 |
| 32 | 2 |

All components were identified using the EEGLAB extended Infomax Independent Component Analysis (ICA) algorithm (Delorme et al., 2007). Independent Components for eye blinks and lateral eye movements were identified for rejection and subtracted from the data by visual inspection of the component scalp topography, time series, and power spectrum distributions. Note that, unusually, three components were removed from the data of nine participants (participants 6, 9, 12, 14, 15, 21, 28, 29 and 30). This was done because the ICA algorithm split one of the two typical eye artifact components (one for eye blink and one for lateral eye movements) into two different components. For example, ICA on participant 9’s data resulted in a duplication of the typical eye blink component. This is probably due to the limited number of six EEG channels used for ICA decomposition. To evaluate whether this limitation would influence the present results, we reproduced the current statistical procedure (cf. Equation 1 described in the Method section) on EEG data without ICA decomposition and corresponding artifact correction. The results of these analyses are reported in Supplementary Table 10.

**Supplementary Table 5 Custom-coded contrast matrix assigned to the Condition predictor of each model.**

| **Condition labels** | **Intercept** | **Exp *vs.* Control**  **(1^st^ contrast)** | **5 *Hz* *vs.* 1 *Hz***  **(2^nd^ contrast)** | **10 *Hz* *vs.* 5 *Hz***  **(3^rd^ contrast)** |
| --- | --- | --- | --- | --- |
| Control | 1 | -3/4 | 0 | 0 |
| 1 *Hz* | 1 | 1/4 | -2/3 | -1/3 |
| 5 *Hz* | 1 | 1/4 | 1/3 | -1/3 |
| 10 *Hz* | 1 | 1/4 | 1/3 | 2/3 |

Within the four conditions composing our task, we manipulate the presence (three Exp) or absence (Control) of the continuous modification of the circle size. We also manipulate, depending on the experimental condition, the frequency rate of this continuous modification: 1 *Hz*, 5 *Hz*, or 10 *Hz*. To include both predictors (i.e., the continuous modification of the circle size, and the frequency at which the circle was modified) in our models and relate to our hypotheses testing, we applied the present custom contrast matrix to the categorical predictor ‘Condition’. This matrix was obtained by applying the generalised inverse to a *Hypothesis* matrix referring to our hypotheses. The ‘Exp *vs.* Control (1^st^ contrast)’ column refers to the hypothesis that there is a difference in spectral power when participants are presented with a continuously modified circle (Exp), relative to when the circle remains the same (Control). The ‘5 *Hz* *vs.* 1 *Hz* (2^nd^ contrast)’ column relates to the hypothesis that there is a difference in spectral power when participants are presented a circle modified at 5 *Hz* relative to 1 *Hz*. The ’10 *Hz* *vs.* 5 *Hz* (3^rd^ contrast)’ column relates to the hypothesis that there is a difference in spectral power when participants are presented a circle modified at 10 *Hz* relative to 5 *Hz*.

**Supplementary Table 6 Custom-coded contrast matrix assigned to the Source predictor of each model.**

| **Source labels** | **Intercept** | **Contrast** |
| --- | --- | --- |
| Online alpha | 1/2 | -0.5 |
| Offline alpha | 1/2 | 0.5 |

When comparing our present study to the one of Maaz et al. (2025), we manipulate the source of the continuous modification of the circle size. The source was, in the current study, the Pz alpha power of the participant computed in real time (Online alpha), and, in Maaz et al. (2025), alpha power computed on prerecorded EEG data. The present matrix was obtained by applying the generalised inverse to a *Hypothesis* matrix referring to the null hypothesis (*H_0_*) regarding the effect of the source of feedback update (i.e., on average, the sum of the dependent variable within each experiment is null).

**Supplementary Table 7 Estimates of the trial repetition effect within the control condition.**

| **Frequency Band** | **Electrode** | **Estimate** | **Lower** | **Upper** | ***BF_10_*** | ***BF_10+_*** |  |
| --- | --- | --- | --- | --- | --- | --- | --- |
| Theta | Fz | 0,01 | 0,004 | 0,017 | 0.329 | > 100 |  |
| **Alpha** | **Fz** | **0,016** | **0,01** | **0,022** | **> 100** | **> 100** |  |
| SMR | Fz | 0,006 | 0 | 0,012 | 0.02 | 36.418 |  |
| Beta | Fz | 0,013 | 0,005 | 0,022 | 0.68 | > 100 |  |
| Theta | Cz | 0,008 | 0,001 | 0,015 | 0.045 | 80.367 |  |
| **Alpha** | **Cz** | **0,017** | **0,011** | **0,023** | **> 100** | **> 100** |  |
| SMR | Cz | 0,003 | -0,002 | 0,009 | 0.006 | 8.98 |  |
| Beta | Cz | 0 | -0,007 | 0,006 | 0.003 | 0.846 |  |
| Theta | Pz | 0,01 | 0,003 | 0,018 | 0.128 | > 100 |  |
| **Alpha** | **Pz** | **0,018** | **0,011** | **0,024** | **> 100** | **> 100** |  |
| SMR | Pz | 0,004 | -0,001 | 0,009 | 0.007 | 12.163 |  |
| Beta | Pz | -0,006 | -0,014 | 0,001 | 0.014 | 0.058 |  |

Each model reported has been computed three times to ensure the stability of the BFs. If not specified, each numerical value corresponds to the average of the values obtained across these three model computations. The ‘Estimate’ column stands for the estimated group-level effect (slope) of the ‘Trial’ predictor considered in a model (in z-score standardised units). The ‘Lower’ and ‘Upper’ columns correspond to the minimal lower and maximal upper bounds of the three 95% CrI computed. The ‘*BF_10_*’ and ‘*BF_10+_*’ columns correspond to the BF in favour of the alternative hypothesis (relative to the null) and the directional (i.e., one-sided) BF, respectively.

Lines in bold highlight the EEG features for which BFs quantify sufficient evidence in favour of the alternative hypothesis over the null (i.e., presence of an effect).

**Supplementary Table 8 Estimates from statistical models computed with Equation 1.**

| **Frequency Band** | **Electrode** | **Predictor** | **Estimate** | **Lower** | **Upper** | ***BF_10_*** | ***BF_10+_*** |
| --- | --- | --- | --- | --- | --- | --- | --- |
| Theta | Fz | Trial | 0,01 | 0,004 | 0,017 | 0.329 | > 100 |
| Theta | Fz | Exp *vs.* Control | 0,038 | -0,026 | 0,102 | 0.065 | 7.315 |
| Theta | Fz | Condition - 5 Hz *vs.* 1 Hz | 0,02 | -0,054 | 0,095 | 0.043 | 2.409 |
| Theta | Fz | Condition - 10 Hz *vs.* 5 Hz | 0,037 | -0,038 | 0,112 | 0.062 | 5.147 |
| Theta | Fz | Source | 0 | -0,24 | 0,244 | 0.121 | 1 |
| Theta | Fz | Trial:Exp *vs.* Control | -0,002 | -0,014 | 0,01 | 0.007 | 0.587 |
| Theta | Fz | Trial:Condition - 5 Hz *vs.* 1 Hz | 0,001 | -0,014 | 0,016 | 0.008 | 1.263 |
| Theta | Fz | Trial:Condition - 10 Hz *vs.* 5 Hz | -0,011 | -0,025 | 0,003 | 0.024 | 0.063 |
| Theta | Fz | Trial:Source | 0 | -0,007 | 0,007 | 0.003 | 0.996 |
| Theta | Fz | Exp *vs.* Control:Source | 0,065 | 0,001 | 0,13 | 0.238 | 42.214 |
| Theta | Fz | Condition - 5 Hz *vs.* 1 Hz:Source | -0,025 | -0,099 | 0,05 | 0.047 | 0.344 |
| Theta | Fz | Condition - 10 Hz *vs.* 5 Hz:Source | 0,003 | -0,072 | 0,079 | 0.039 | 1.15 |
| Theta | Fz | Trial:Exp *vs.* Control:Source | -0,008 | -0,02 | 0,004 | 0.015 | 0.102 |
| Theta | Fz | Trial:Condition - 5 Hz *vs.* 1 Hz:Source | 0,006 | -0,009 | 0,02 | 0.01 | 3.501 |
| Theta | Fz | Trial:Condition - 10 Hz *vs.* 5 Hz:Source | -0,007 | -0,021 | 0,007 | 0.012 | 0.182 |
| **Alpha** | **Fz** | **Trial** | **0,016** | **0,01** | **0,022** | **> 100** | **> 100** |
| Alpha | Fz | Exp *vs.* Control | 0,03 | -0,031 | 0,09 | 0.049 | 5.059 |
| Alpha | Fz | Condition - 5 Hz *vs.* 1 Hz | -0,005 | -0,072 | 0,062 | 0.034 | 0.797 |
| Alpha | Fz | Condition - 10 Hz *vs.* 5 Hz | 0,053 | -0,025 | 0,132 | 0.098 | 10.126 |
| Alpha | Fz | Source | -0,011 | -0,259 | 0,236 | 0.125 | 0.869 |
| Alpha | Fz | Trial:Exp *vs.* Control | -0,001 | -0,012 | 0,01 | 0.006 | 0.778 |
| Alpha | Fz | Trial:Condition - 5 Hz *vs.* 1 Hz | -0,007 | -0,02 | 0,007 | 0.011 | 0.191 |
| Alpha | Fz | Trial:Condition - 10 Hz *vs.* 5 Hz | 0,004 | -0,009 | 0,018 | 0.008 | 2.787 |
| Alpha | Fz | Trial:Source | 0,003 | -0,002 | 0,009 | 0.006 | 7.259 |
| Alpha | Fz | Exp *vs.* Control:Source | -0,012 | -0,073 | 0,049 | 0.033 | 0.536 |
| Alpha | Fz | Condition - 5 Hz *vs.* 1 Hz:Source | -0,001 | -0,067 | 0,066 | 0.033 | 0.958 |
| Alpha | Fz | Condition - 10 Hz *vs.* 5 Hz:Source | -0,04 | -0,119 | 0,039 | 0.067 | 0.188 |
| Alpha | Fz | Trial:Exp *vs.* Control:Source | 0,006 | -0,005 | 0,017 | 0.01 | 5.817 |
| Alpha | Fz | Trial:Condition - 5 Hz *vs.* 1 Hz:Source | -0,003 | -0,016 | 0,011 | 0.007 | 0.529 |
| Alpha | Fz | Trial:Condition - 10 Hz *vs.* 5 Hz:Source | 0,001 | -0,013 | 0,014 | 0.007 | 1.18 |
| SMR | Fz | Trial | 0,006 | 0 | 0,012 | 0.02 | 36.418 |
| SMR | Fz | Exp *vs.* Control | 0,091 | 0,031 | 0,152 | 2.338 | > 100 |
| SMR | Fz | Condition - 5 Hz *vs.* 1 Hz | -0,065 | -0,134 | 0,003 | 0.206 | 0.03 |
| SMR | Fz | Condition - 10 Hz *vs.* 5 Hz | 0,073 | -0,001 | 0,146 | 0.249 | 37.479 |
| SMR | Fz | Source | -0,014 | -0,265 | 0,24 | 0.128 | 0.834 |
| SMR | Fz | Trial:Exp *vs.* Control | -0,006 | -0,018 | 0,007 | 0.009 | 0.218 |
| SMR | Fz | Trial:Condition - 5 Hz *vs.* 1 Hz | 0,01 | -0,006 | 0,025 | 0.017 | 8.348 |
| SMR | Fz | Trial:Condition - 10 Hz *vs.* 5 Hz | -0,001 | -0,016 | 0,014 | 0.008 | 0.854 |
| SMR | Fz | Trial:Source | 0,004 | -0,002 | 0,01 | 0.007 | 10.216 |
| SMR | Fz | Exp *vs.* Control:Source | 0,012 | -0,048 | 0,072 | 0.032 | 1.855 |
| SMR | Fz | Condition - 5 Hz *vs.* 1 Hz:Source | -0,043 | -0,111 | 0,025 | 0.075 | 0.117 |
| SMR | Fz | Condition - 10 Hz *vs.* 5 Hz:Source | -0,033 | -0,107 | 0,04 | 0.056 | 0.225 |
| SMR | Fz | Trial:Exp *vs.* Control:Source | 0,001 | -0,011 | 0,013 | 0.006 | 1.238 |
| SMR | Fz | Trial:Condition - 5 Hz *vs.* 1 Hz:Source | 0,007 | -0,008 | 0,023 | 0.012 | 4.708 |
| SMR | Fz | Trial:Condition - 10 Hz *vs.* 5 Hz:Source | -0,003 | -0,018 | 0,012 | 0.008 | 0.487 |
| Beta | Fz | Trial | 0,013 | 0,005 | 0,022 | 0.68 | > 100 |
| Beta | Fz | Exp *vs.* Control | 0,085 | -0,006 | 0,176 | 0.258 | 29.654 |
| Beta | Fz | Condition - 5 Hz *vs.* 1 Hz | -0,031 | -0,139 | 0,077 | 0.064 | 0.398 |
| Beta | Fz | Condition - 10 Hz *vs.* 5 Hz | 0,098 | -0,007 | 0,203 | 0.292 | 29.473 |
| Beta | Fz | Source | -0,017 | -0,258 | 0,229 | 0.123 | 0.8 |
| Beta | Fz | Trial:Exp *vs.* Control | -0,003 | -0,019 | 0,013 | 0.009 | 0.533 |
| Beta | Fz | Trial:Condition - 5 Hz *vs.* 1 Hz | -0,002 | -0,024 | 0,021 | 0.011 | 0.787 |
| Beta | Fz | Trial:Condition - 10 Hz *vs.* 5 Hz | 0,004 | -0,016 | 0,024 | 0.011 | 1.846 |
| Beta | Fz | Trial:Source | 0,005 | -0,003 | 0,013 | 0.008 | 6.894 |
| Beta | Fz | Exp *vs.* Control:Source | 0,002 | -0,089 | 0,092 | 0.045 | 1.061 |
| Beta | Fz | Condition - 5 Hz *vs.* 1 Hz:Source | -0,023 | -0,13 | 0,086 | 0.059 | 0.509 |
| Beta | Fz | Condition - 10 Hz *vs.* 5 Hz:Source | -0,019 | -0,123 | 0,086 | 0.056 | 0.567 |
| Beta | Fz | Trial:Exp *vs.* Control:Source | 0,002 | -0,014 | 0,018 | 0.008 | 1.585 |
| Beta | Fz | Trial:Condition - 5 Hz *vs.* 1 Hz:Source | 0,001 | -0,022 | 0,023 | 0.011 | 1.105 |
| Beta | Fz | Trial:Condition - 10 Hz *vs.* 5 Hz:Source | 0 | -0,02 | 0,02 | 0.01 | 0.973 |
| Theta | Cz | Trial | 0,008 | 0,001 | 0,015 | 0.045 | 80.367 |
| Theta | Cz | Exp *vs.* Control | 0,074 | 0,018 | 0,131 | 0.755 | > 100 |
| Theta | Cz | Condition - 5 Hz *vs.* 1 Hz | 0,024 | -0,051 | 0,1 | 0.047 | 2.844 |
| Theta | Cz | Condition - 10 Hz *vs.* 5 Hz | 0,035 | -0,038 | 0,108 | 0.057 | 4.762 |
| Theta | Cz | Source | -0,001 | -0,243 | 0,24 | 0.12 | 0.986 |
| Theta | Cz | Trial:Exp *vs.* Control | -0,004 | -0,017 | 0,009 | 0.008 | 0.374 |
| Theta | Cz | Trial:Condition - 5 Hz *vs.* 1 Hz | 0,003 | -0,012 | 0,018 | 0.008 | 1.853 |
| Theta | Cz | Trial:Condition - 10 Hz *vs.* 5 Hz | -0,013 | -0,027 | 0,001 | 0.037 | 0.037 |
| Theta | Cz | Trial:Source | 0 | -0,006 | 0,007 | 0.003 | 1.226 |
| Theta | Cz | Exp *vs.* Control:Source | 0,074 | 0,017 | 0,131 | 0.713 | > 100 |
| Theta | Cz | Condition - 5 Hz *vs.* 1 Hz:Source | 0,01 | -0,066 | 0,085 | 0.039 | 1.528 |
| Theta | Cz | Condition - 10 Hz *vs.* 5 Hz:Source | -0,017 | -0,09 | 0,056 | 0.041 | 0.476 |
| Theta | Cz | Trial:Exp *vs.* Control:Source | -0,007 | -0,02 | 0,006 | 0.011 | 0.179 |
| Theta | Cz | Trial:Condition - 5 Hz *vs.* 1 Hz:Source | 0,002 | -0,013 | 0,016 | 0.008 | 1.39 |
| Theta | Cz | Trial:Condition - 10 Hz *vs.* 5 Hz:Source | -0,006 | -0,021 | 0,008 | 0.011 | 0.236 |
| **Alpha** | **Cz** | **Trial** | **0,017** | **0,011** | **0,023** | **> 100** | **> 100** |
| Alpha | Cz | Exp *vs.* Control | 0,014 | -0,047 | 0,075 | 0.034 | 2.07 |
| Alpha | Cz | Condition - 5 Hz *vs.* 1 Hz | -0,012 | -0,084 | 0,059 | 0.038 | 0.585 |
| Alpha | Cz | Condition - 10 Hz *vs.* 5 Hz | 0,051 | -0,025 | 0,126 | 0.091 | 9.871 |
| Alpha | Cz | Source | -0,02 | -0,263 | 0,225 | 0.123 | 0.768 |
| Alpha | Cz | Trial:Exp *vs.* Control | 0 | -0,012 | 0,011 | 0.006 | 0.875 |
| Alpha | Cz | Trial:Condition - 5 Hz *vs.* 1 Hz | -0,006 | -0,02 | 0,008 | 0.011 | 0.23 |
| Alpha | Cz | Trial:Condition - 10 Hz *vs.* 5 Hz | 0 | -0,014 | 0,014 | 0.007 | 1 |
| Alpha | Cz | Trial:Source | 0,006 | 0 | 0,012 | 0.017 | 29.384 |
| Alpha | Cz | Exp *vs.* Control:Source | -0,018 | -0,079 | 0,043 | 0.037 | 0.391 |
| Alpha | Cz | Condition - 5 Hz *vs.* 1 Hz:Source | 0,002 | -0,069 | 0,073 | 0.036 | 1.098 |
| Alpha | Cz | Condition - 10 Hz *vs.* 5 Hz:Source | -0,053 | -0,128 | 0,022 | 0.101 | 0.089 |
| Alpha | Cz | Trial:Exp *vs.* Control:Source | 0,003 | -0,009 | 0,015 | 0.007 | 2.303 |
| Alpha | Cz | Trial:Condition - 5 Hz *vs.* 1 Hz:Source | -0,004 | -0,018 | 0,01 | 0.008 | 0.434 |
| Alpha | Cz | Trial:Condition - 10 Hz *vs.* 5 Hz:Source | 0,004 | -0,01 | 0,017 | 0.008 | 2.38 |
| SMR | Cz | Trial | 0,003 | -0,002 | 0,009 | 0.006 | 8.98 |
| SMR | Cz | Exp *vs.* Control | 0,077 | 0,017 | 0,138 | 0.707 | > 100 |
| SMR | Cz | Condition - 5 Hz *vs.* 1 Hz | -0,042 | -0,103 | 0,019 | 0.077 | 0.095 |
| SMR | Cz | Condition - 10 Hz *vs.* 5 Hz | 0,076 | 0,01 | 0,142 | 0.432 | 82.487 |
| SMR | Cz | Source | -0,008 | -0,263 | 0,249 | 0.128 | 0.9 |
| SMR | Cz | Trial:Exp *vs.* Control | -0,005 | -0,018 | 0,009 | 0.009 | 0.324 |
| SMR | Cz | Trial:Condition - 5 Hz *vs.* 1 Hz | 0,006 | -0,01 | 0,021 | 0.01 | 3.351 |
| SMR | Cz | Trial:Condition - 10 Hz *vs.* 5 Hz | -0,002 | -0,017 | 0,012 | 0.007 | 0.603 |
| SMR | Cz | Trial:Source | 0,002 | -0,003 | 0,007 | 0.003 | 3.42 |
| SMR | Cz | Exp *vs.* Control:Source | 0,019 | -0,041 | 0,079 | 0.037 | 2.765 |
| SMR | Cz | Condition - 5 Hz *vs.* 1 Hz:Source | -0,036 | -0,097 | 0,025 | 0.062 | 0.138 |
| SMR | Cz | Condition - 10 Hz *vs.* 5 Hz:Source | -0,021 | -0,087 | 0,045 | 0.041 | 0.355 |
| SMR | Cz | Trial:Exp *vs.* Control:Source | 0,001 | -0,012 | 0,014 | 0.007 | 1.299 |
| SMR | Cz | Trial:Condition - 5 Hz *vs.* 1 Hz:Source | 0,006 | -0,009 | 0,021 | 0.011 | 3.693 |
| SMR | Cz | Trial:Condition - 10 Hz *vs.* 5 Hz:Source | -0,009 | -0,023 | 0,005 | 0.016 | 0.114 |
| Beta | Cz | Trial | 0 | -0,007 | 0,006 | 0.003 | 0.846 |
| Beta | Cz | Exp *vs.* Control | 0,053 | -0,027 | 0,133 | 0.097 | 9.446 |
| Beta | Cz | Condition - 5 Hz *vs.* 1 Hz | -0,02 | -0,082 | 0,042 | 0.039 | 0.355 |
| Beta | Cz | Condition - 10 Hz *vs.* 5 Hz | 0,105 | 0,022 | 0,187 | 0.95 | > 100 |
| Beta | Cz | Source | -0,004 | -0,254 | 0,249 | 0.126 | 0.957 |
| Beta | Cz | Trial:Exp *vs.* Control | 0 | -0,015 | 0,015 | 0.008 | 1.015 |
| Beta | Cz | Trial:Condition - 5 Hz *vs.* 1 Hz | -0,005 | -0,02 | 0,011 | 0.01 | 0.369 |
| Beta | Cz | Trial:Condition - 10 Hz *vs.* 5 Hz | 0 | -0,017 | 0,017 | 0.009 | 1.007 |
| Beta | Cz | Trial:Source | 0,001 | -0,005 | 0,008 | 0.004 | 2.008 |
| Beta | Cz | Exp *vs.* Control:Source | -0,023 | -0,104 | 0,057 | 0.048 | 0.388 |
| Beta | Cz | Condition - 5 Hz *vs.* 1 Hz:Source | -0,046 | -0,108 | 0,015 | 0.093 | 0.075 |
| Beta | Cz | Condition - 10 Hz *vs.* 5 Hz:Source | 0,01 | -0,073 | 0,092 | 0.043 | 1.44 |
| Beta | Cz | Trial:Exp *vs.* Control:Source | 0,008 | -0,008 | 0,023 | 0.013 | 5.299 |
| Beta | Cz | Trial:Condition - 5 Hz *vs.* 1 Hz:Source | 0,006 | -0,01 | 0,021 | 0.01 | 3.207 |
| Beta | Cz | Trial:Condition - 10 Hz *vs.* 5 Hz:Source | -0,007 | -0,024 | 0,01 | 0.012 | 0.255 |
| Theta | Pz | Trial | 0,01 | 0,003 | 0,018 | 0.128 | > 100 |
| Theta | Pz | Exp *vs.* Control | 0,081 | 0,021 | 0,141 | 1.059 | > 100 |
| Theta | Pz | Condition - 5 Hz *vs.* 1 Hz | 0,01 | -0,074 | 0,094 | 0.043 | 1.432 |
| Theta | Pz | Condition - 10 Hz *vs.* 5 Hz | 0,007 | -0,069 | 0,083 | 0.039 | 1.334 |
| Theta | Pz | Source | -0,001 | -0,238 | 0,24 | 0.118 | 0.985 |
| Theta | Pz | Trial:Exp *vs.* Control | -0,006 | -0,019 | 0,007 | 0.01 | 0.235 |
| Theta | Pz | Trial:Condition - 5 Hz *vs.* 1 Hz | 0,003 | -0,013 | 0,019 | 0.009 | 1.732 |
| Theta | Pz | Trial:Condition - 10 Hz *vs.* 5 Hz | -0,011 | -0,026 | 0,004 | 0.023 | 0.072 |
| Theta | Pz | Trial:Source | 0 | -0,008 | 0,008 | 0.004 | 0.997 |
| Theta | Pz | Exp *vs.* Control:Source | 0,085 | 0,025 | 0,145 | 1.457 | > 100 |
| Theta | Pz | Condition - 5 Hz *vs.* 1 Hz:Source | -0,014 | -0,098 | 0,07 | 0.045 | 0.592 |
| Theta | Pz | Condition - 10 Hz *vs.* 5 Hz:Source | -0,042 | -0,118 | 0,034 | 0.07 | 0.162 |
| Theta | Pz | Trial:Exp *vs.* Control:Source | -0,01 | -0,023 | 0,004 | 0.019 | 0.087 |
| Theta | Pz | Trial:Condition - 5 Hz *vs.* 1 Hz:Source | 0,003 | -0,013 | 0,02 | 0.009 | 1.941 |
| Theta | Pz | Trial:Condition - 10 Hz *vs.* 5 Hz:Source | -0,005 | -0,02 | 0,009 | 0.01 | 0.302 |
| **Alpha** | **Pz** | **Trial** | **0,018** | **0,011** | **0,024** | **> 100** | **> 100** |
| Alpha | Pz | Exp *vs.* Control | -0,02 | -0,088 | 0,047 | 0.041 | 0.378 |
| Alpha | Pz | Condition - 5 Hz *vs.* 1 Hz | -0,01 | -0,079 | 0,058 | 0.036 | 0.619 |
| Alpha | Pz | Condition - 10 Hz *vs.* 5 Hz | 0,036 | -0,043 | 0,115 | 0.06 | 4.503 |
| Alpha | Pz | Source | -0,022 | -0,265 | 0,22 | 0.123 | 0.748 |
| Alpha | Pz | Trial:Exp *vs.* Control | -0,005 | -0,017 | 0,008 | 0.008 | 0.312 |
| Alpha | Pz | Trial:Condition - 5 Hz *vs.* 1 Hz | -0,007 | -0,021 | 0,007 | 0.012 | 0.181 |
| Alpha | Pz | Trial:Condition - 10 Hz *vs.* 5 Hz | 0 | -0,013 | 0,013 | 0.007 | 0.961 |
| Alpha | Pz | Trial:Source | 0,006 | 0 | 0,013 | 0.023 | 41.899 |
| Alpha | Pz | Exp *vs.* Control:Source | 0,005 | -0,063 | 0,073 | 0.034 | 1.285 |
| Alpha | Pz | Condition - 5 Hz *vs.* 1 Hz:Source | 0,008 | -0,061 | 0,076 | 0.035 | 1.422 |
| Alpha | Pz | Condition - 10 Hz *vs.* 5 Hz:Source | -0,015 | -0,095 | 0,063 | 0.043 | 0.537 |
| Alpha | Pz | Trial:Exp *vs.* Control:Source | 0,002 | -0,011 | 0,014 | 0.007 | 1.474 |
| Alpha | Pz | Trial:Condition - 5 Hz *vs.* 1 Hz:Source | -0,007 | -0,021 | 0,007 | 0.012 | 0.187 |
| Alpha | Pz | Trial:Condition - 10 Hz *vs.* 5 Hz:Source | 0 | -0,013 | 0,014 | 0.007 | 1.098 |
| SMR | Pz | Trial | 0,004 | -0,001 | 0,009 | 0.007 | 12.163 |
| SMR | Pz | Exp *vs.* Control | 0,068 | 0,007 | 0,128 | 0.354 | 70.559 |
| SMR | Pz | Condition - 5 Hz *vs.* 1 Hz | -0,023 | -0,08 | 0,034 | 0.039 | 0.271 |
| SMR | Pz | Condition - 10 Hz *vs.* 5 Hz | 0,039 | -0,021 | 0,099 | 0.07 | 8.925 |
| SMR | Pz | Source | -0,003 | -0,262 | 0,253 | 0.128 | 0.957 |
| SMR | Pz | Trial:Exp *vs.* Control | -0,008 | -0,02 | 0,003 | 0.017 | 0.078 |
| SMR | Pz | Trial:Condition - 5 Hz *vs.* 1 Hz | 0 | -0,015 | 0,015 | 0.007 | 0.955 |
| SMR | Pz | Trial:Condition - 10 Hz *vs.* 5 Hz | 0,001 | -0,012 | 0,014 | 0.007 | 1.235 |
| SMR | Pz | Trial:Source | 0,001 | -0,004 | 0,006 | 0.003 | 2.057 |
| SMR | Pz | Exp *vs.* Control:Source | 0,024 | -0,037 | 0,084 | 0.042 | 3.661 |
| SMR | Pz | Condition - 5 Hz *vs.* 1 Hz:Source | -0,014 | -0,071 | 0,043 | 0.033 | 0.462 |
| SMR | Pz | Condition - 10 Hz *vs.* 5 Hz:Source | -0,026 | -0,086 | 0,034 | 0.044 | 0.242 |
| SMR | Pz | Trial:Exp *vs.* Control:Source | 0,002 | -0,009 | 0,014 | 0.006 | 1.808 |
| SMR | Pz | Trial:Condition - 5 Hz *vs.* 1 Hz:Source | -0,001 | -0,015 | 0,014 | 0.007 | 0.861 |
| SMR | Pz | Trial:Condition - 10 Hz *vs.* 5 Hz:Source | -0,003 | -0,016 | 0,01 | 0.008 | 0.443 |
| Beta | Pz | Trial | -0,006 | -0,014 | 0,001 | 0.014 | 0.058 |
| Beta | Pz | Exp *vs.* Control | 0,063 | -0,027 | 0,154 | 0.121 | 10.839 |
| Beta | Pz | Condition - 5 Hz *vs.* 1 Hz | -0,013 | -0,073 | 0,046 | 0.033 | 0.485 |
| Beta | Pz | Condition - 10 Hz *vs.* 5 Hz | 0,09 | -0,002 | 0,182 | 0.298 | 35.523 |
| Beta | Pz | Source | 0,003 | -0,252 | 0,258 | 0.127 | 1.036 |
| Beta | Pz | Trial:Exp *vs.* Control | -0,001 | -0,018 | 0,016 | 0.009 | 0.838 |
| Beta | Pz | Trial:Condition - 5 Hz *vs.* 1 Hz | -0,011 | -0,026 | 0,004 | 0.021 | 0.079 |
| Beta | Pz | Trial:Condition - 10 Hz *vs.* 5 Hz | -0,001 | -0,019 | 0,018 | 0.009 | 0.87 |
| Beta | Pz | Trial:Source | -0,001 | -0,008 | 0,006 | 0.004 | 0.635 |
| Beta | Pz | Exp *vs.* Control:Source | 0,006 | -0,085 | 0,097 | 0.046 | 1.223 |
| Beta | Pz | Condition - 5 Hz *vs.* 1 Hz:Source | -0,02 | -0,079 | 0,04 | 0.038 | 0.343 |
| Beta | Pz | Condition - 10 Hz *vs.* 5 Hz:Source | 0,019 | -0,074 | 0,11 | 0.049 | 1.908 |
| Beta | Pz | Trial:Exp *vs.* Control:Source | 0,007 | -0,01 | 0,025 | 0.012 | 4.092 |
| Beta | Pz | Trial:Condition - 5 Hz *vs.* 1 Hz:Source | -0,003 | -0,018 | 0,011 | 0.008 | 0.489 |
| Beta | Pz | Trial:Condition - 10 Hz *vs.* 5 Hz:Source | -0,007 | -0,025 | 0,012 | 0.012 | 0.301 |

Each model reported has been computed three times in order to ensure the stability of the BFs. If not specified, each numerical value corresponds to the average of the values obtained across these three model computations. The ‘Estimate’ column stands for the estimated group-level effect (slope) of each predictor considered in a model (in z-score standardised units). For the ‘Trial’ predictor, the estimate corresponds to the group-level effect of one trial of the control condition (modality of Condition predictor defined as reference for subsequent comparisons) and averaged across both experiments (defined as reference for subsequent comparisons for the Source predictor). For the ‘Exp *vs.* Control’, ‘5 *Hz* *vs.* 1 *Hz*’ and ‘10 *Hz* *vs.* 5 *Hz*’ predictors, the estimate refers to the group-level effect when comparing, during each condition first trial (modality of Trial predictor defined as reference for subsequent comparisons) and averaged across both experiments (defined as reference for subsequent comparisons for the Source predictor), the mean of the three Experimental conditions (1 *Hz*, 5 *Hz*, 10 *Hz*) to the control condition, the 5 *Hz* Condition to the 1 *Hz* Condition, and the 10 *Hz* Condition to the 1 *Hz* Condition, respectively. Concerning the ‘Source’ predictor, the estimate refers to the group-level effect, during the first trial (modality of Trial predictor defined as reference for subsequent comparisons) of the control condition (modality of Condition predictor defined as reference for subsequent comparisons), when comparing the group for which the source of the feedback update was Online alpha (current experiment) and the group for which it was Offline alpha (experiment of Maaz et al., 2025). The ‘Lower’ and ‘Upper’ columns correspond to the minimal lower and maximal upper bounds of the three 95% CrI computed. The ‘*BF_10_*’ and ‘*BF_10+_*’ columns correspond to the BF in favour of the alternative hypothesis (relative to the null) and the directional (i.e., one-sided) BF, respectively.

Lines in gold highlight the EEG features for which BFs quantify sufficient evidence in favour of the alternative hypothesis over the null (i.e., presence of an effect).

**Supplementary Table 9 Estimates from models considering the ‘Trial’ predictor (integers of 1 to 32) throughout the entire task.**

| **Frequency Band** | **Electrode** | **Parameter** | **Estimate** | **Lower** | **Upper** | ***BF_10_*** | ***BF_10+_*** |
| --- | --- | --- | --- | --- | --- | --- | --- |
| Theta | Fz | Trial | 0,006 | 0,003 | 0,009 | 0.792 | > 100 |
| Theta | Fz | Source | 0,025 | -0,207 | 0,255 | 0.119 | 1.433 |
| Theta | Fz | Trial:Source | -0,002 | -0,005 | 0,001 | 0.003 | 0.161 |
| **Alpha** | **Fz** | **Trial** | **0,007** | **0,004** | **0,01** | **> 100** | **> 100** |
| Alpha | Fz | Source | 0,041 | -0,188 | 0,269 | 0.122 | 1.772 |
| Alpha | Fz | Trial:Source | -0,003 | -0,005 | 0 | 0.009 | 0.027 |
| SMR | Fz | Trial | 0,003 | 0,001 | 0,005 | 0.026 | > 100 |
| SMR | Fz | Source | 0,026 | -0,212 | 0,266 | 0.122 | 1.413 |
| SMR | Fz | Trial:Source | -0,002 | -0,004 | 0,001 | 0.003 | 0.101 |
| Beta | Fz | Trial | 0,005 | 0,002 | 0,008 | 0.121 | > 100 |
| Beta | Fz | Source | -0,012 | -0,243 | 0,221 | 0.115 | 0.843 |
| Beta | Fz | Trial:Source | 0,001 | -0,002 | 0,004 | 0.002 | 2.164 |
| Theta | Cz | Trial | 0,003 | 0 | 0,006 | 0.011 | 34.99 |
| Theta | Cz | Source | 0,021 | -0,217 | 0,255 | 0.12 | 1.333 |
| Theta | Cz | Trial:Source | -0,001 | -0,005 | 0,002 | 0.002 | 0.23 |
| **Alpha** | **Cz** | **Trial** | **0,007** | **0,004** | **0,01** | **> 100** | **> 100** |
| Alpha | Cz | Source | 0,057 | -0,167 | 0,28 | 0.129 | 2.293 |
| Alpha | Cz | Trial:Source | -0,004 | -0,007 | -0,001 | 0.04 | 0.005 |
| SMR | Cz | Trial | 0,002 | 0 | 0,004 | 0.005 | 24.922 |
| SMR | Cz | Source | 0,019 | -0,227 | 0,262 | 0.123 | 1.284 |
| SMR | Cz | Trial:Source | -0,001 | -0,003 | 0,001 | 0.002 | 0.137 |
| Beta | Cz | Trial | 0,001 | -0,001 | 0,004 | 0.002 | 6.62 |
| Beta | Cz | Source | -0,003 | -0,245 | 0,24 | 0.121 | 0.965 |
| Beta | Cz | Trial:Source | 0 | -0,002 | 0,003 | 0.001 | 1.266 |
| Theta | Pz | Trial | 0,006 | 0,002 | 0,009 | 0.253 | > 100 |
| Theta | Pz | Source | 0,037 | -0,183 | 0,26 | 0.118 | 1.689 |
| Theta | Pz | Trial:Source | -0,002 | -0,006 | 0,001 | 0.004 | 0.092 |
| **Alpha** | **Pz** | **Trial** | **0,009** | **0,006** | **0,012** | **> 100** | **> 100** |
| Alpha | Pz | Source | 0,072 | -0,154 | 0,296 | 0.138 | 2.812 |
| Alpha | Pz | Trial:Source | -0,005 | -0,007 | -0,002 | 0.473 | 0 |
| SMR | Pz | Trial | 0,002 | 0 | 0,004 | 0.014 | 80.223 |
| SMR | Pz | Source | 0,022 | -0,226 | 0,27 | 0.127 | 1.327 |
| SMR | Pz | Trial:Source | -0,001 | -0,004 | 0,001 | 0.003 | 0.085 |
| Beta | Pz | Trial | 0 | -0,003 | 0,003 | 0.001 | 0.874 |
| Beta | Pz | Source | -0,001 | -0,245 | 0,245 | 0.122 | 0.98 |
| Beta | Pz | Trial:Source | 0 | -0,002 | 0,003 | 0.001 | 1.191 |

Each model reported has been computed three times in order to ensure the stability of the BFs. If not specified, each numerical value corresponds to the average of the values obtained across these three model computations. The ‘Estimate’ column stands for the estimated group-level effects (slopes) of each model ‘Parameter’ (in z-score standardised units). For the ‘Trial’ predictor, the estimate corresponds to the group-level effect of one trial averaged across both experiments (defined as reference for subsequent comparisons for the Source predictor). For the ‘Source’ predictor, the estimate refers to the group-level effect, during the first trial of the session (modality of Trial predictor defined as reference for subsequent comparisons), when comparing the group for which the source of the feedback update was Online alpha (current experiment) and the group for which it was Offline alpha (experiment of Maaz et al., 2025). The ‘Lower’ and ‘Upper’ columns correspond to the minimal lower and maximal upper bounds of the three 95% CrI computed. The ‘*BF_10_*’ and ‘*BF_10+_*’ columns correspond to the BF in favour of the alternative hypothesis (relative to the null) and the directional (i.e., one-sided) BF, respectively.

Lines in gold highlight the EEG features for which BFs quantify sufficient evidence in favour of the alternative hypothesis over the null (i.e., presence of an effect).

**Supplementary Table 10 Estimates from models computed with Equation 1 on EEG data without ICA-based artifact correction.**

| **Frequency Band** | **Electrode** | **Predictor** | **Estimate** | **Lower** | **Upper** | ***BF_10_*** | ***BF_10+_*** |
| --- | --- | --- | --- | --- | --- | --- | --- |
| Theta | Fz | Trial | 0,002 | -0,011 | 0,014 | 0.007 | 1.461 |
| Theta | Fz | Exp *vs.* Control | -0,141 | -0,259 | -0,024 | 0.992 | 0.01 |
| Theta | Fz | Condition - 5 Hz *vs.* 1 Hz | 0 | -0,123 | 0,124 | 0.062 | 1.014 |
| **Theta** | **Fz** | **Condition - 10 Hz *vs.* 5 Hz** | **0,172** | **0,063** | **0,28** | **6.424** | **> 100** |
| Theta | Fz | Source | -0,013 | -0,244 | 0,219 | 0.114 | 0.839 |
| Theta | Fz | Trial:Exp *vs.* Control | 0,017 | -0,003 | 0,038 | 0.042 | 20.05 |
| Theta | Fz | Trial:Condition - 5 Hz *vs.* 1 Hz | -0,003 | -0,025 | 0,019 | 0.012 | 0.615 |
| Theta | Fz | Trial:Condition - 10 Hz *vs.* 5 Hz | -0,02 | -0,045 | 0,005 | 0.046 | 0.058 |
| Theta | Fz | Trial:Source | 0,004 | -0,009 | 0,017 | 0.008 | 2.727 |
| Theta | Fz | Exp *vs.* Control:Source | 0,02 | -0,098 | 0,137 | 0.062 | 1.714 |
| Theta | Fz | Condition - 5 Hz *vs.* 1 Hz:Source | -0,033 | -0,156 | 0,091 | 0.072 | 0.428 |
| Theta | Fz | Condition - 10 Hz *vs.* 5 Hz:Source | 0,075 | -0,033 | 0,183 | 0.138 | 10.674 |
| Theta | Fz | Trial:Exp *vs.* Control:Source | 0,006 | -0,015 | 0,026 | 0.012 | 2.479 |
| Theta | Fz | Trial:Condition - 5 Hz *vs.* 1 Hz:Source | 0,011 | -0,011 | 0,033 | 0.018 | 5.514 |
| Theta | Fz | Trial:Condition - 10 Hz *vs.* 5 Hz:Source | -0,009 | -0,034 | 0,016 | 0.016 | 0.304 |
| Alpha | Fz | Trial | 0,013 | 0,006 | 0,021 | 1.261 | > 100 |
| Alpha | Fz | Exp *vs.* Control | -0,018 | -0,096 | 0,06 | 0.044 | 0.478 |
| Alpha | Fz | Condition - 5 Hz *vs.* 1 Hz | -0,026 | -0,11 | 0,06 | 0.052 | 0.382 |
| Alpha | Fz | Condition - 10 Hz *vs.* 5 Hz | 0,125 | 0,038 | 0,213 | 2.152 | > 100 |
| Alpha | Fz | Source | -0,003 | -0,243 | 0,237 | 0.119 | 0.958 |
| Alpha | Fz | Trial:Exp *vs.* Control | 0,008 | -0,005 | 0,022 | 0.014 | 7.75 |
| Alpha | Fz | Trial:Condition - 5 Hz *vs.* 1 Hz | -0,001 | -0,017 | 0,015 | 0.008 | 0.768 |
| Alpha | Fz | Trial:Condition - 10 Hz *vs.* 5 Hz | -0,004 | -0,02 | 0,012 | 0.009 | 0.444 |
| Alpha | Fz | Trial:Source | 0,001 | -0,007 | 0,008 | 0.004 | 1.254 |
| Alpha | Fz | Exp *vs.* Control:Source | 0,047 | -0,031 | 0,125 | 0.081 | 7.716 |
| Alpha | Fz | Condition - 5 Hz *vs.* 1 Hz:Source | 0,028 | -0,058 | 0,113 | 0.054 | 2.913 |
| Alpha | Fz | Condition - 10 Hz *vs.* 5 Hz:Source | 0,044 | -0,043 | 0,132 | 0.074 | 5.374 |
| Alpha | Fz | Trial:Exp *vs.* Control:Source | -0,007 | -0,021 | 0,006 | 0.012 | 0.168 |
| Alpha | Fz | Trial:Condition - 5 Hz *vs.* 1 Hz:Source | 0,003 | -0,013 | 0,018 | 0.008 | 1.672 |
| Alpha | Fz | Trial:Condition - 10 Hz *vs.* 5 Hz:Source | -0,002 | -0,018 | 0,014 | 0.008 | 0.671 |
| SMR | Fz | Trial | 0,002 | -0,005 | 0,009 | 0.004 | 2.386 |
| SMR | Fz | Exp *vs.* Control | 0,095 | 0,009 | 0,182 | 0.467 | 62.509 |
| SMR | Fz | Condition - 5 Hz *vs.* 1 Hz | -0,058 | -0,154 | 0,037 | 0.102 | 0.127 |
| **SMR** | **Fz** | **Condition - 10 Hz *vs.* 5 Hz** | **0,14** | **0,053** | **0,226** | **5.664** | **> 100** |
| SMR | Fz | Source | 0,005 | -0,245 | 0,254 | 0.125 | 1.073 |
| SMR | Fz | Trial:Exp *vs.* Control | -0,007 | -0,023 | 0,009 | 0.012 | 0.244 |
| SMR | Fz | Trial:Condition - 5 Hz *vs.* 1 Hz | 0,01 | -0,009 | 0,029 | 0.017 | 5.752 |
| SMR | Fz | Trial:Condition - 10 Hz *vs.* 5 Hz | -0,005 | -0,024 | 0,014 | 0.011 | 0.453 |
| SMR | Fz | Trial:Source | -0,002 | -0,009 | 0,005 | 0.004 | 0.445 |
| SMR | Fz | Exp *vs.* Control:Source | 0,005 | -0,082 | 0,091 | 0.044 | 1.207 |
| SMR | Fz | Condition - 5 Hz *vs.* 1 Hz:Source | 0,051 | -0,044 | 0,146 | 0.085 | 5.888 |
| SMR | Fz | Condition - 10 Hz *vs.* 5 Hz:Source | 0,054 | -0,034 | 0,14 | 0.092 | 7.978 |
| SMR | Fz | Trial:Exp *vs.* Control:Source | 0 | -0,017 | 0,016 | 0.008 | 0.929 |
| SMR | Fz | Trial:Condition - 5 Hz *vs.* 1 Hz:Source | -0,007 | -0,027 | 0,012 | 0.013 | 0.297 |
| SMR | Fz | Trial:Condition - 10 Hz *vs.* 5 Hz:Source | 0,005 | -0,015 | 0,024 | 0.011 | 2.211 |
| Beta | Fz | Trial | 0 | -0,014 | 0,012 | 0.007 | 0.879 |
| Beta | Fz | Exp *vs.* Control | 0,126 | -0,003 | 0,255 | 0.413 | 36.136 |
| Beta | Fz | Condition - 5 Hz *vs.* 1 Hz | -0,021 | -0,158 | 0,117 | 0.073 | 0.615 |
| **Beta** | **Fz** | **Condition - 10 Hz *vs.* 5 Hz** | **0,201** | **0,081** | **0,32** | **11.197** | **> 100** |
| Beta | Fz | Source | -0,007 | -0,24 | 0,228 | 0.117 | 0.903 |
| Beta | Fz | Trial:Exp *vs.* Control | -0,008 | -0,033 | 0,016 | 0.016 | 0.332 |
| Beta | Fz | Trial:Condition - 5 Hz *vs.* 1 Hz | 0 | -0,026 | 0,027 | 0.013 | 1.041 |
| Beta | Fz | Trial:Condition - 10 Hz *vs.* 5 Hz | -0,003 | -0,028 | 0,022 | 0.013 | 0.708 |
| Beta | Fz | Trial:Source | 0,002 | -0,011 | 0,015 | 0.007 | 1.689 |
| Beta | Fz | Exp *vs.* Control:Source | 0,05 | -0,079 | 0,18 | 0.087 | 3.514 |
| Beta | Fz | Condition - 5 Hz *vs.* 1 Hz:Source | 0,048 | -0,09 | 0,185 | 0.087 | 3.101 |
| Beta | Fz | Condition - 10 Hz *vs.* 5 Hz:Source | 0,022 | -0,097 | 0,142 | 0.064 | 1.808 |
| Beta | Fz | Trial:Exp *vs.* Control:Source | -0,01 | -0,035 | 0,014 | 0.018 | 0.252 |
| Beta | Fz | Trial:Condition - 5 Hz *vs.* 1 Hz:Source | -0,002 | -0,029 | 0,024 | 0.014 | 0.737 |
| Beta | Fz | Trial:Condition - 10 Hz *vs.* 5 Hz:Source | 0,012 | -0,014 | 0,037 | 0.019 | 4.492 |
| Theta | Cz | Trial | 0,01 | 0 | 0,019 | 0.038 | 44.684 |
| Theta | Cz | Exp *vs.* Control | 0,007 | -0,073 | 0,088 | 0.041 | 1.324 |
| Theta | Cz | Condition - 5 Hz *vs.* 1 Hz | 0,014 | -0,089 | 0,117 | 0.054 | 1.548 |
| Theta | Cz | Condition - 10 Hz *vs.* 5 Hz | 0,094 | 0,013 | 0,174 | 0.579 | 87.489 |
| Theta | Cz | Source | -0,017 | -0,251 | 0,217 | 0.116 | 0.794 |
| Theta | Cz | Trial:Exp *vs.* Control | 0,002 | -0,015 | 0,02 | 0.009 | 1.559 |
| Theta | Cz | Trial:Condition - 5 Hz *vs.* 1 Hz | 0,002 | -0,017 | 0,021 | 0.01 | 1.348 |
| Theta | Cz | Trial:Condition - 10 Hz *vs.* 5 Hz | -0,013 | -0,031 | 0,006 | 0.024 | 0.092 |
| Theta | Cz | Trial:Source | 0,005 | -0,005 | 0,014 | 0.008 | 5.66 |
| Theta | Cz | Exp *vs.* Control:Source | -0,041 | -0,122 | 0,039 | 0.067 | 0.184 |
| Theta | Cz | Condition - 5 Hz *vs.* 1 Hz:Source | 0 | -0,103 | 0,103 | 0.052 | 0.992 |
| Theta | Cz | Condition - 10 Hz *vs.* 5 Hz:Source | 0,014 | -0,066 | 0,094 | 0.042 | 1.722 |
| Theta | Cz | Trial:Exp *vs.* Control:Source | 0,006 | -0,011 | 0,024 | 0.011 | 3.177 |
| Theta | Cz | Trial:Condition - 5 Hz *vs.* 1 Hz:Source | 0,002 | -0,016 | 0,021 | 0.01 | 1.5 |
| Theta | Cz | Trial:Condition - 10 Hz *vs.* 5 Hz:Source | 0,002 | -0,017 | 0,02 | 0.01 | 1.409 |
| **Alpha** | **Cz** | **Trial** | **0,02** | **0,013** | **0,026** | **> 100** | **> 100** |
| Alpha | Cz | Exp *vs.* Control | 0,003 | -0,065 | 0,071 | 0.034 | 1.139 |
| Alpha | Cz | Condition - 5 Hz *vs.* 1 Hz | -0,005 | -0,083 | 0,074 | 0.04 | 0.835 |
| Alpha | Cz | Condition - 10 Hz *vs.* 5 Hz | 0,068 | -0,013 | 0,15 | 0.165 | 19.574 |
| Alpha | Cz | Source | 0,005 | -0,237 | 0,244 | 0.122 | 1.066 |
| Alpha | Cz | Trial:Exp *vs.* Control | 0,001 | -0,012 | 0,013 | 0.006 | 1.183 |
| Alpha | Cz | Trial:Condition - 5 Hz *vs.* 1 Hz | -0,008 | -0,023 | 0,008 | 0.013 | 0.192 |
| Alpha | Cz | Trial:Condition - 10 Hz *vs.* 5 Hz | 0,001 | -0,013 | 0,016 | 0.007 | 1.364 |
| Alpha | Cz | Trial:Source | -0,002 | -0,009 | 0,005 | 0.004 | 0.48 |
| Alpha | Cz | Exp *vs.* Control:Source | 0,019 | -0,05 | 0,087 | 0.04 | 2.476 |
| Alpha | Cz | Condition - 5 Hz *vs.* 1 Hz:Source | 0,031 | -0,048 | 0,109 | 0.053 | 3.524 |
| Alpha | Cz | Condition - 10 Hz *vs.* 5 Hz:Source | 0,026 | -0,056 | 0,107 | 0.051 | 2.728 |
| Alpha | Cz | Trial:Exp *vs.* Control:Source | -0,004 | -0,017 | 0,009 | 0.008 | 0.384 |
| Alpha | Cz | Trial:Condition - 5 Hz *vs.* 1 Hz:Source | 0,002 | -0,013 | 0,018 | 0.008 | 1.541 |
| Alpha | Cz | Trial:Condition - 10 Hz *vs.* 5 Hz:Source | -0,001 | -0,015 | 0,014 | 0.007 | 0.831 |
| SMR | Cz | Trial | 0,003 | -0,003 | 0,009 | 0.004 | 4.184 |
| SMR | Cz | Exp *vs.* Control | 0,088 | 0,01 | 0,166 | 0.466 | 72.992 |
| SMR | Cz | Condition - 5 Hz *vs.* 1 Hz | -0,035 | -0,115 | 0,045 | 0.059 | 0.242 |
| SMR | Cz | Condition - 10 Hz *vs.* 5 Hz | 0,12 | 0,04 | 0,199 | 2.902 | > 100 |
| SMR | Cz | Source | 0,007 | -0,248 | 0,264 | 0.126 | 1.091 |
| SMR | Cz | Trial:Exp *vs.* Control | -0,007 | -0,023 | 0,009 | 0.012 | 0.246 |
| SMR | Cz | Trial:Condition - 5 Hz *vs.* 1 Hz | 0,006 | -0,011 | 0,023 | 0.011 | 3.135 |
| SMR | Cz | Trial:Condition - 10 Hz *vs.* 5 Hz | -0,003 | -0,02 | 0,015 | 0.009 | 0.621 |
| SMR | Cz | Trial:Source | -0,002 | -0,008 | 0,004 | 0.004 | 0.378 |
| SMR | Cz | Exp *vs.* Control:Source | -0,015 | -0,093 | 0,062 | 0.042 | 0.544 |
| SMR | Cz | Condition - 5 Hz *vs.* 1 Hz:Source | 0,034 | -0,046 | 0,113 | 0.058 | 3.969 |
| SMR | Cz | Condition - 10 Hz *vs.* 5 Hz:Source | 0,04 | -0,04 | 0,119 | 0.064 | 5.155 |
| SMR | Cz | Trial:Exp *vs.* Control:Source | -0,001 | -0,017 | 0,015 | 0.008 | 0.857 |
| SMR | Cz | Trial:Condition - 5 Hz *vs.* 1 Hz:Source | -0,003 | -0,02 | 0,014 | 0.009 | 0.594 |
| SMR | Cz | Trial:Condition - 10 Hz *vs.* 5 Hz:Source | 0,01 | -0,007 | 0,027 | 0.016 | 6.749 |
| Beta | Cz | Trial | -0,007 | -0,018 | 0,004 | 0.013 | 0.106 |
| Beta | Cz | Exp *vs.* Control | 0,112 | -0,007 | 0,231 | 0.336 | 29.736 |
| Beta | Cz | Condition - 5 Hz *vs.* 1 Hz | 0,019 | -0,081 | 0,118 | 0.053 | 1.812 |
| **Beta** | **Cz** | **Condition - 10 Hz *vs.* 5 Hz** | **0,157** | **0,05** | **0,264** | **3.144** | **> 100** |
| Beta | Cz | Source | 0,002 | -0,241 | 0,245 | 0.122 | 1.039 |
| Beta | Cz | Trial:Exp *vs.* Control | -0,008 | -0,032 | 0,016 | 0.016 | 0.32 |
| Beta | Cz | Trial:Condition - 5 Hz *vs.* 1 Hz | -0,005 | -0,026 | 0,016 | 0.012 | 0.49 |
| Beta | Cz | Trial:Condition - 10 Hz *vs.* 5 Hz | 0 | -0,022 | 0,023 | 0.012 | 1.072 |
| Beta | Cz | Trial:Source | -0,001 | -0,012 | 0,01 | 0.005 | 0.724 |
| Beta | Cz | Exp *vs.* Control:Source | 0,076 | -0,044 | 0,196 | 0.133 | 8.578 |
| Beta | Cz | Condition - 5 Hz *vs.* 1 Hz:Source | 0,09 | -0,01 | 0,189 | 0.244 | 25.166 |
| Beta | Cz | Condition - 10 Hz *vs.* 5 Hz:Source | 0 | -0,107 | 0,108 | 0.054 | 1.012 |
| Beta | Cz | Trial:Exp *vs.* Control:Source | -0,016 | -0,04 | 0,008 | 0.028 | 0.104 |
| Beta | Cz | Trial:Condition - 5 Hz *vs.* 1 Hz:Source | -0,011 | -0,032 | 0,01 | 0.018 | 0.184 |
| Beta | Cz | Trial:Condition - 10 Hz *vs.* 5 Hz:Source | 0,016 | -0,007 | 0,039 | 0.031 | 11.098 |
| Theta | Pz | Trial | 0,013 | 0,003 | 0,023 | 0.149 | > 100 |
| Theta | Pz | Exp *vs.* Control | 0,046 | -0,038 | 0,13 | 0.076 | 6.248 |
| Theta | Pz | Condition - 5 Hz *vs.* 1 Hz | 0,005 | -0,108 | 0,119 | 0.058 | 1.156 |
| Theta | Pz | Condition - 10 Hz *vs.* 5 Hz | 0,059 | -0,028 | 0,146 | 0.108 | 10.029 |
| Theta | Pz | Source | -0,016 | -0,243 | 0,211 | 0.114 | 0.782 |
| Theta | Pz | Trial:Exp *vs.* Control | -0,003 | -0,021 | 0,015 | 0.009 | 0.582 |
| Theta | Pz | Trial:Condition - 5 Hz *vs.* 1 Hz | -0,002 | -0,022 | 0,018 | 0.01 | 0.734 |
| Theta | Pz | Trial:Condition - 10 Hz *vs.* 5 Hz | -0,007 | -0,027 | 0,012 | 0.013 | 0.297 |
| Theta | Pz | Trial:Source | 0,005 | -0,005 | 0,015 | 0.008 | 4.975 |
| Theta | Pz | Exp *vs.* Control:Source | -0,068 | -0,153 | 0,017 | 0.154 | 0.06 |
| Theta | Pz | Condition - 5 Hz *vs.* 1 Hz:Source | 0,028 | -0,086 | 0,141 | 0.064 | 2.184 |
| Theta | Pz | Condition - 10 Hz *vs.* 5 Hz:Source | 0,028 | -0,058 | 0,116 | 0.054 | 2.875 |
| Theta | Pz | Trial:Exp *vs.* Control:Source | 0,01 | -0,007 | 0,028 | 0.017 | 6.866 |
| Theta | Pz | Trial:Condition - 5 Hz *vs.* 1 Hz:Source | 0,003 | -0,017 | 0,023 | 0.011 | 1.558 |
| Theta | Pz | Trial:Condition - 10 Hz *vs.* 5 Hz:Source | 0 | -0,019 | 0,02 | 0.01 | 1.079 |
| **Alpha** | **Pz** | **Trial** | **0,021** | **0,014** | **0,028** | **> 100** | **> 100** |
| Alpha | Pz | Exp *vs.* Control | -0,042 | -0,119 | 0,035 | 0.069 | 0.166 |
| Alpha | Pz | Condition - 5 Hz *vs.* 1 Hz | -0,012 | -0,094 | 0,07 | 0.043 | 0.627 |
| Alpha | Pz | Condition - 10 Hz *vs.* 5 Hz | 0,049 | -0,035 | 0,132 | 0.083 | 6.986 |
| Alpha | Pz | Source | 0,01 | -0,225 | 0,246 | 0.117 | 1.149 |
| Alpha | Pz | Trial:Exp *vs.* Control | -0,004 | -0,018 | 0,01 | 0.008 | 0.417 |
| Alpha | Pz | Trial:Condition - 5 Hz *vs.* 1 Hz | -0,008 | -0,024 | 0,008 | 0.013 | 0.184 |
| Alpha | Pz | Trial:Condition - 10 Hz *vs.* 5 Hz | -0,001 | -0,015 | 0,014 | 0.007 | 0.897 |
| Alpha | Pz | Trial:Source | -0,003 | -0,01 | 0,004 | 0.005 | 0.269 |
| Alpha | Pz | Exp *vs.* Control:Source | -0,013 | -0,09 | 0,064 | 0.041 | 0.58 |
| Alpha | Pz | Condition - 5 Hz *vs.* 1 Hz:Source | 0,004 | -0,078 | 0,085 | 0.042 | 1.148 |
| Alpha | Pz | Condition - 10 Hz *vs.* 5 Hz:Source | 0,008 | -0,076 | 0,092 | 0.042 | 1.334 |
| Alpha | Pz | Trial:Exp *vs.* Control:Source | -0,004 | -0,018 | 0,01 | 0.008 | 0.401 |
| Alpha | Pz | Trial:Condition - 5 Hz *vs.* 1 Hz:Source | 0,009 | -0,007 | 0,024 | 0.014 | 6.242 |
| Alpha | Pz | Trial:Condition - 10 Hz *vs.* 5 Hz:Source | 0,001 | -0,014 | 0,015 | 0.007 | 1.127 |
| SMR | Pz | Trial | 0,004 | -0,003 | 0,01 | 0.006 | 6.044 |
| SMR | Pz | Exp *vs.* Control | 0,089 | 0,013 | 0,163 | 0.557 | 90.818 |
| SMR | Pz | Condition - 5 Hz *vs.* 1 Hz | -0,043 | -0,127 | 0,042 | 0.071 | 0.186 |
| SMR | Pz | Condition - 10 Hz *vs.* 5 Hz | 0,073 | -0,002 | 0,148 | 0.238 | 35.532 |
| SMR | Pz | Source | 0,005 | -0,246 | 0,253 | 0.126 | 1.067 |
| SMR | Pz | Trial:Exp *vs.* Control | -0,013 | -0,027 | 0 | 0.048 | 0.024 |
| SMR | Pz | Trial:Condition - 5 Hz *vs.* 1 Hz | 0,003 | -0,014 | 0,021 | 0.01 | 1.761 |
| SMR | Pz | Trial:Condition - 10 Hz *vs.* 5 Hz | 0,002 | -0,015 | 0,018 | 0.009 | 1.393 |
| SMR | Pz | Trial:Source | -0,002 | -0,008 | 0,005 | 0.004 | 0.45 |
| SMR | Pz | Exp *vs.* Control:Source | -0,006 | -0,081 | 0,069 | 0.038 | 0.782 |
| SMR | Pz | Condition - 5 Hz *vs.* 1 Hz:Source | -0,023 | -0,108 | 0,061 | 0.049 | 0.42 |
| SMR | Pz | Condition - 10 Hz *vs.* 5 Hz:Source | 0,052 | -0,022 | 0,127 | 0.101 | 11.223 |
| SMR | Pz | Trial:Exp *vs.* Control:Source | -0,006 | -0,02 | 0,007 | 0.011 | 0.205 |
| SMR | Pz | Trial:Condition - 5 Hz *vs.* 1 Hz:Source | 0,012 | -0,006 | 0,029 | 0.021 | 9.518 |
| SMR | Pz | Trial:Condition - 10 Hz *vs.* 5 Hz:Source | 0,002 | -0,015 | 0,018 | 0.008 | 1.333 |
| Beta | Pz | Trial | -0,013 | -0,024 | -0,002 | 0.088 | 0.01 |
| Beta | Pz | Exp *vs.* Control | 0,142 | 0,01 | 0,274 | 0.627 | 56.566 |
| Beta | Pz | Condition - 5 Hz *vs.* 1 Hz | -0,022 | -0,14 | 0,097 | 0.064 | 0.557 |
| **Beta** | **Pz** | **Condition - 10 Hz *vs.* 5 Hz** | **0,184** | **0,067** | **0,3** | **6.253** | **> 100** |
| Beta | Pz | Source | 0,007 | -0,238 | 0,251 | 0.12 | 1.111 |
| Beta | Pz | Trial:Exp *vs.* Control | -0,014 | -0,038 | 0,011 | 0.022 | 0.16 |
| Beta | Pz | Trial:Condition - 5 Hz *vs.* 1 Hz | -0,004 | -0,028 | 0,021 | 0.013 | 0.609 |
| Beta | Pz | Trial:Condition - 10 Hz *vs.* 5 Hz | -0,007 | -0,03 | 0,017 | 0.014 | 0.41 |
| Beta | Pz | Trial:Source | -0,002 | -0,013 | 0,009 | 0.006 | 0.529 |
| Beta | Pz | Exp *vs.* Control:Source | 0,073 | -0,059 | 0,206 | 0.124 | 6.431 |
| Beta | Pz | Condition - 5 Hz *vs.* 1 Hz:Source | 0,015 | -0,105 | 0,134 | 0.061 | 1.504 |
| Beta | Pz | Condition - 10 Hz *vs.* 5 Hz:Source | 0,012 | -0,103 | 0,128 | 0.061 | 1.401 |
| Beta | Pz | Trial:Exp *vs.* Control:Source | -0,019 | -0,043 | 0,006 | 0.039 | 0.072 |
| Beta | Pz | Trial:Condition - 5 Hz *vs.* 1 Hz:Source | 0,002 | -0,022 | 0,027 | 0.012 | 1.348 |
| Beta | Pz | Trial:Condition - 10 Hz *vs.* 5 Hz:Source | 0,012 | -0,011 | 0,036 | 0.021 | 5.638 |

Each model reported has been computed twice in order to ensure the stability of the BFs. If not specified, each numerical value corresponds to the average of the values obtained across these two model computations. The ‘Estimate’ column stands for the estimated group-level effect (slope) of each predictor considered in a model (in z-score standardised units). For the ‘Trial’ predictor, the estimate corresponds to the group-level effect of one trial of the control condition (modality of Condition predictor defined as reference for subsequent comparisons) and averaged across both experiments (defined as reference for subsequent comparisons for the Source predictor). For the ‘Exp *vs.* Control’, ‘5 *Hz* *vs.* 1 *Hz*’ and ‘10 *Hz* *vs.* 5 *Hz*’ predictors, the estimate refers to the group-level effect when comparing, during each condition first trial (modality of Trial predictor defined as reference for subsequent comparisons) and averaged across both experiments (defined as reference for subsequent comparisons for the Source predictor), the mean of the three Experimental conditions (1 *Hz*, 5 *Hz*, 10 *Hz*) to the control condition, the 5 *Hz* Condition to the 1 *Hz* Condition, and the 10 *Hz* Condition to the 1 *Hz* Condition, respectively. Concerning the ‘Source’ predictor, the estimate refers to the group-level effect, during the first trial (modality of Trial predictor defined as reference for subsequent comparisons) of the control condition (modality of Condition predictor defined as reference for subsequent comparisons), when comparing the group for which the source of the feedback update was Online alpha (current experiment) and the group for which it was Offline alpha (experiment of Maaz et al., 2025). The ‘Lower’ and ‘Upper’ columns correspond to the minimal lower and maximal upper bounds of the two 95% CrI computed. The ‘*BF_10_*’ and ‘*BF_10+_*’ columns correspond to the BF in favour of the alternative hypothesis (relative to the null) and the directional (i.e., one-sided) BF, respectively.

Lines in gold highlight the EEG features for which BFs quantify sufficient evidence in favour of the alternative hypothesis over the null (i.e., presence of an effect).

Comparatively to the results obtained on EEG data with ICA-based artifact correction (cf. Supplementary Tables 7-8), few differences must be acknowledged. Here, substantial Bayesian evidence was quantified for an effect of the frequency at which the circle was modified on the spectral power of theta (Fz), SMR (Fz) and beta (Fz, Cz and Pz) bands. Additionally, insensitive amount of evidence was found concerning the effect of Trial repetition on alpha power at Pz. This particularly contrasts with the extreme level of evidence reported on the data on which ocular artifacts were removed after ICA decomposition (cf. Supplementary Tables 7-8). However, more importantly, the main results of alpha increases with Trial repetition at Cz and Pz were consistently reproduced. Thus, given that the discrepancies in results almost exclusively occurred on Fz data, this could be explained by the greater spatial proximity of Fz electrode with the eyes and the absence of ocular-artifact correction in the present analyses.

**
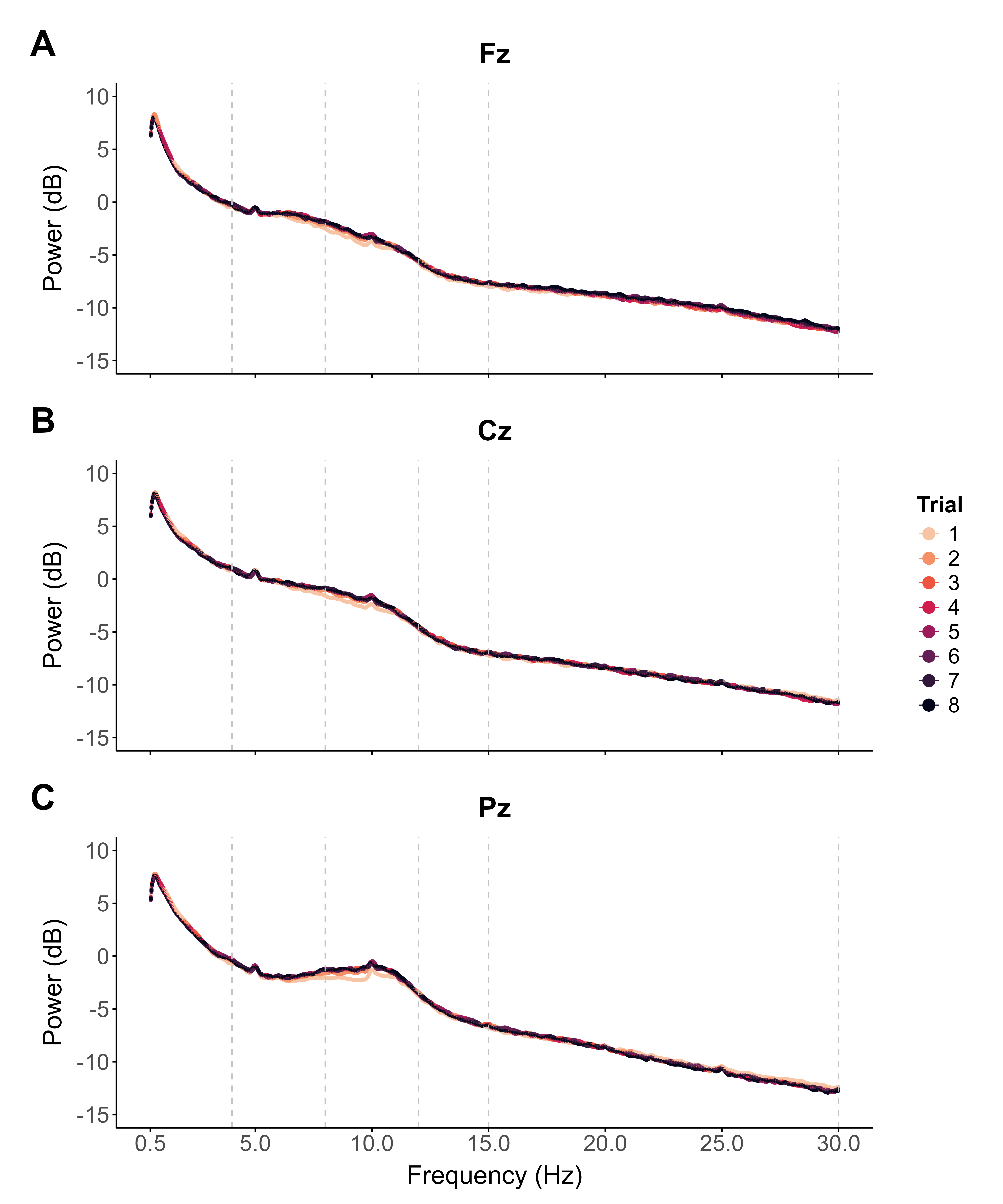
Supplementary Figure 1 Averaged power spectra for each trial.** Power spectra depending on the trial number (from clearer to darker colours: 1:8) and on electrode position (A: Fz; B: Cz; C: Pz). Each spectrum has been obtained by averaging spectral power across participants and conditions with a frequency resolution of ~0.305 *Hz*. On each panel, vertical dashed lines represent the boundaries each of the frequency bands considered as dependent variables, i.e., theta (4-8 *Hz*), alpha (8-12 *Hz*), SMR (12-15 *Hz*) and beta (15-30 *Hz*). Consistently with the statistical results (see Supplementary Table 8), substantial increases in power are solely observed within the alpha range for each electrode.

**
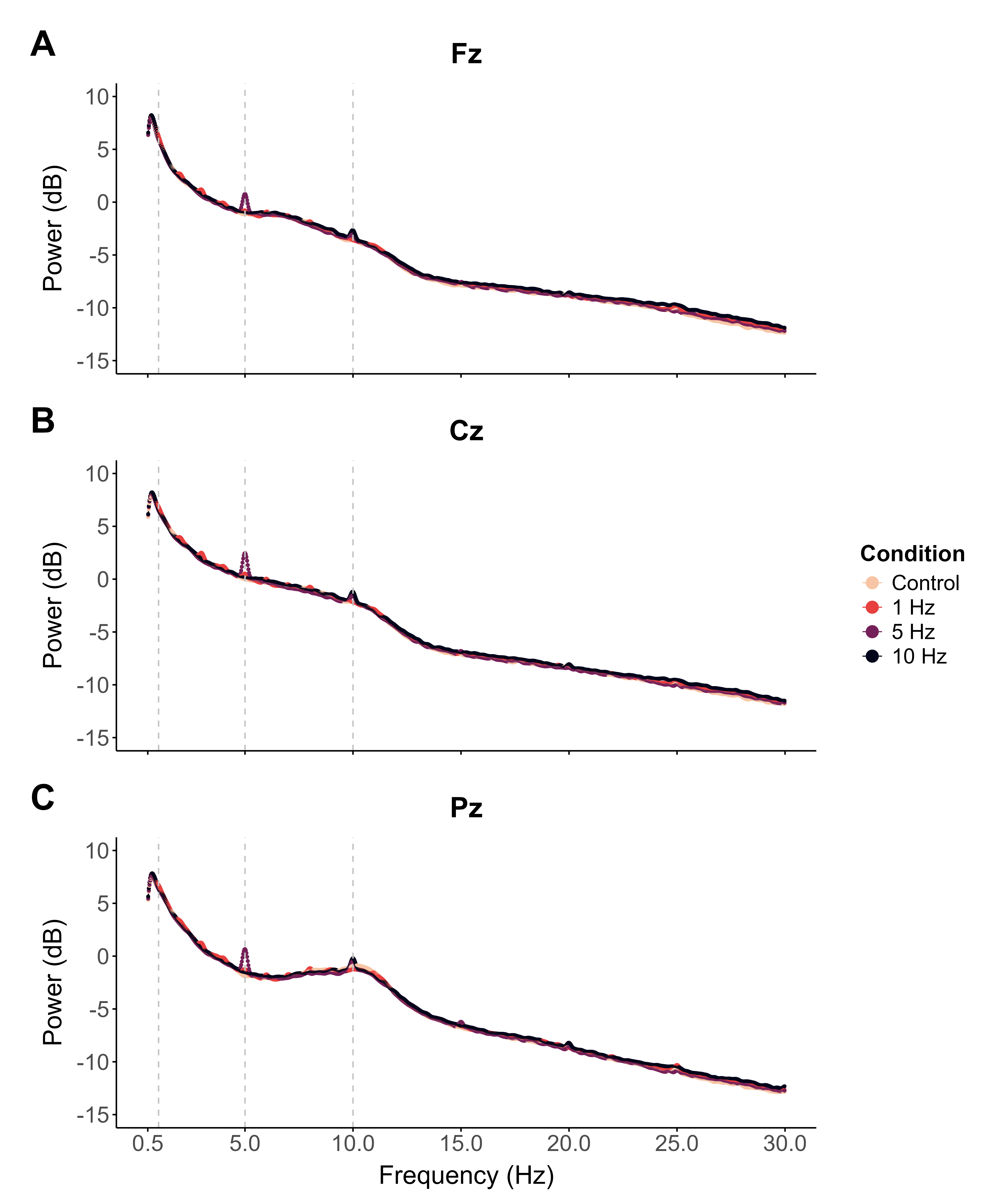
Supplementary Figure 2 Averaged power spectra depending on the presence of continuous circle size modification and its corresponding frequency rate.** Power spectra depending on the condition (cream: control – no circle modification; red: 1 *Hz*; violet: 5 *Hz*; dark blue: 10 *Hz*) and on electrode position (A: Fz; B: Cz; C: Pz). Each spectrum has been obtained by averaging spectral power across participants and trials with a frequency resolution of ~0.305 *Hz*. On each panel, vertical dashed lines represent each of the feedback update frequencies.

For each electrode, noticeable power peaks appear at 5 *Hz* (i.e., within the theta range of 4-8 *Hz*) and at 10 *Hz* (i.e., within the alpha range of 8-12 *Hz*) during the 5 *Hz* and the 10 *Hz* conditions, respectively, compared to the others. However, statistically, these peaks are not strong enough to substantially increase spectral densities when computed over corresponding frequency bands (i.e., theta and alpha, respectively; see Supplementary Table 8).
